# A multilevel layout algorithm for visualizing physical and genetic interaction networks, with emphasis on their modular organization

**DOI:** 10.1186/1756-0381-5-2

**Published:** 2012-03-26

**Authors:** Johannes Tuikkala, Heidi Vähämaa, Pekka Salmela, Olli S Nevalainen, Tero Aittokallio

**Affiliations:** 1 Department of Information Technology, FI-20014 University of Turku, Turku, Finland; 2 Department of Mathematics, FI-20014 University of Turku, Turku, Finland; 3 Institute for Molecular Medicine Finland (FIMM), FI-00014 University of Helsinki, Helsinki, Finland

## Abstract

**Background:**

Graph drawing is an integral part of many systems biology studies, enabling visual exploration and mining of large-scale biological networks. While a number of layout algorithms are available in popular network analysis platforms, such as Cytoscape, it remains poorly understood how well their solutions reflect the underlying biological processes that give rise to the network connectivity structure. Moreover, visualizations obtained using conventional layout algorithms, such as those based on the force-directed drawing approach, may become uninformative when applied to larger networks with dense or clustered connectivity structure.

**Methods:**

We implemented a modified layout plug-in, named Multilevel Layout, which applies the conventional layout algorithms within a multilevel optimization framework to better capture the hierarchical modularity of many biological networks. Using a wide variety of real life biological networks, we carried out a systematic evaluation of the method in comparison with other layout algorithms in Cytoscape.

**Results:**

The multilevel approach provided both biologically relevant and visually pleasant layout solutions in most network types, hence complementing the layout options available in Cytoscape. In particular, it could improve drawing of large-scale networks of yeast genetic interactions and human physical interactions. In more general terms, the biological evaluation framework developed here enables one to assess the layout solutions from any existing or future graph drawing algorithm as well as to optimize their performance for a given network type or structure.

**Conclusions:**

By making use of the multilevel modular organization when visualizing biological networks, together with the biological evaluation of the layout solutions, one can generate convenient visualizations for many network biology applications.

## Background

Network graphs provide a valuable conceptual framework for representing and mining high-throughput experimental datasets, as well as for extracting and interpreting their biological information by the means of graph-based analysis approaches [1-8]. In cellular systems, network nodes typically refer to biomolecules, such as genes or proteins, and the edge connections the type of relationships the network is encoding, including physical or functional information. Network visualization aims to organize the complex network structures in a way that provides the user with readily apparent insights into the most interesting biological patterns and relationships within the data, such as components of biological pathways, processes or complexes, which can be further investigated by follow-up computational and/or experimental analyses [4-6,9,10]. Owing to the developments in biotechnologies, experimental datasets are steadily increasing in their size and complexity, posing many challenges to the network-centric data visualization and biological exploration.

There exists a wide variety of advanced network layout algorithms that seek to place connected nodes of a graph close to each other. Conventionally, these layout algorithms are specifically designed for a particular network type, such as gene regulatory networks or signalling pathways [11,12], metabolic pathways or biochemical networks [13-15], or phylogenetic networks [16]. Algorithmic solutions have also been introduced for specific network topologies, such as drawing fragmented networks [17], grid layouts [18], or detailed visualization of small networks [19]. However, there exists no universal layout solution, and therefore a practical strategy involves trying out multiple layout algorithms a number of times to see which one best arranges a given network [6,20]. Such a test-and-trial strategy often neglects the biological relevance of the layout solutions, as well as requires bioinformatics skills or resources to allow experimenting with several algorithms, many of which are not implemented as user-friendly software packages.

To provide researchers with an easy access to network visualization tools, several network analysis software come with sophisticated methods for laying out networks. Such software packages, each providing a specific range of visualization options, include, e.g., VisANT, NAViGaTOR, PATIKA, PINA, MATISSE, GraphViz, Osprey, Graphle, CellDesigner, Biolayout, ProViz and Pajek; see [4,5,10] and references therein. Among others, Cytoscape software platform for network analysis and visualization has been widely adopted by the biological community because of its ease of use, compatibility with and direct access to many network formats and databases, respectively, as well as straightforward extensibility through open-source plug-in development [4,5,20,21]. In its core, a number of advanced layout algorithms are available, including those based on spring-embedded and force -directed graph drawing approaches [22,23]. Many of these algorithms work reasonably well, especially for small- and medium-sized networks (e.g., 50-1000 nodes), whereas larger networks, in particular those with a dense or clustered connectivity structure, are more difficult to visualize, often resulting in 'hairball' network layouts [4-6].

Many biological networks have shown to represent with a modular organization [24], which often manifests in a hierarchical cluster structure of highly interconnected network modules across a spectrum of resolution levels [1-3]. Such modular architecture has been revealed using both physical mapping of protein interaction networks [1], as well as by quantitative mapping of genetic interactions networks [25]. These two network types encode fundamental and partly complementary information about physical and functional relationships among biomolecules. Protein-protein interaction networks characterize physical relationships between proteins that are in direct binding contact or co-existence in a complex. Changes in the observed modularity of the human protein interaction networks has been used, for instance, to predict biological and clinical outcomes, such as brain cancer progression or breast cancer metastasis [26,27]. Genetic interaction networks mapped by combinations of pairwise gene mutations in model organisms, such as budding yeast, have revealed highly hierarchical maps of inter-connected network modules, such as components of compensatory pathways or protein complexes, and their functional cross-connections that regulate cellular processes and maintain mutational robustness [28,29].

We hypothesized that such a multilevel organization of the network connectivity structure could be utilized to provide both visually attractive and biologically relevant network layouts. Therefore, we implemented a Cytoscape plug-in, named Multilevel Layout, which combines traditional node placement algorithms with a multilevel optimization framework introduced by Walshaw [30]. The multilevel framework first constructs a hierarchy of increasingly coarser graphs and then applies the force-directed placement at each level of resolution to generate globally clear and aesthetic layout solutions. Our implemented version of the generic multilevel framework is modified for network biology applications, e.g., by including options for grouping the nodes based on their degree of connectivity or using clustering coefficient to further emphasize the hierarchical modularity of the networks. We have previously demonstrated that the longer running time of the multilevel approach, compared to the traditional layout options, is compensated by its capacity to provide visually pleasant layouts also for larger networks [31].

In the present work, we investigated whether the multilevel layout approach could provide also biologically meaningful network visualizations, in addition to being visually attractive. To this end, we compared the multilevel layout solutions to those generated by popular layout algorithms in the Cytoscpape platform, in terms of their capacity at capturing the semantic similarity information about underlying biological processes. Based on such systematic comparative evaluations on various large-scale networks, originating both from physical and genetic interaction mappings, one could provide the users with a practical guidance on how to choose a preferable layout algorithm for different network types and their characteristic properties. To facilitate drawing of networks with several thousands of nodes, we improved the computational complexity of the multilevel approach through the use of efficient M-tree database structures. To promote their widespread usage in network biology applications, we have made available efficient implementations of both the Multilevel Layout algorithm and Biological Evaluation as plug-ins for Cytoscape software.

## Methods

### Multilevel layout algorithm

**Figure 1:**
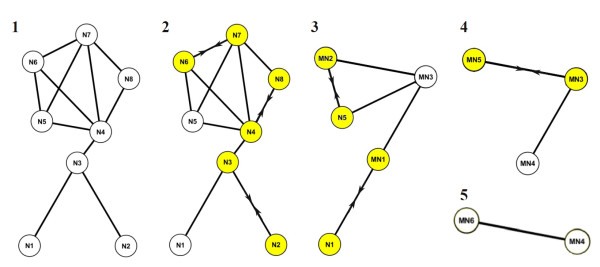
**Graph coarsening for multilevel organization**. The coarsening process is visualized in a sub-network of Ito-Core (8 nodes, 10 edges). The arrows and highlighting indicate those nodes that are combined into a new metanode (MN). At the first level of the coarsening (1), nodes N2 and N3; nodes N4 and N8; and nodes N6 and N7 are merged into metanodes MN1, MN3, and MN2 (2). At the next level (3), node N1 and metanode MN1 are merged into a new metanode MN3; and node N5 and metanode MN2 are merged into metanode MN5. Finally, after merging metanodes MN5 and MN3 into metanode MN6 (4), there are only two nodes left and the coarsening process is finished (5).

The multilevel framework for graph drawing was originally proposed by Walshaw [30]. The generic algorithm combines the traditional force-directed node placement method of Fruchterman and Reingold [23] with multilevel organization by recursively coarsening graphs in two phases. In the first phase, the algorithm generates a set of increasingly coarser graphs, *G*_0_, *G*_1_, ..., *G_L_*, where *G*_0 _is the original graph for which the layout is being calculated, and *G_L _*is the coarsest graph consisting of only two nodes and one connecting edge. The graph *G_i _*is said to be on the level *i *of the graph hierarchy, or the level *i *of the progress of the multilevel algorithm. In the multilevel concept, the graphs are coarsened by finding a maximal independent subset of edges and by combining the nodes connected by these edges into a metanode in the graph on the next level of the hierarchy (Figure 1). Since the problem of solving the maximal independent edge set problem is generally NP-hard, and the computation time is essential for many users of the network biology platforms, we content ourselves here on non-optimal solutions. The coarsening scheme proposed by Walshaw [30] was to pick a random node and match it with a neighboring node with the smallest weight (defined to be 1 for each node in *G*_0 _and the number of original nodes inside a metanode in *G*_i _for *i > 0*). If there are nodes without a suitable matching partner in *G*_*i*-1_, then those nodes form singleton nodes in the graph on the next level *G_i_*. To deal with specific types of biological networks, such as those having 'star-like' structures, we modified the weighting function by taking into account also the node degree (the number of edges incident to the node) in the matching phase [31] (Additional File 1).

In the second phase of the multilevel method, the graphs *G_L_, G*_*L*-1_, ..., *G_1 _*are uncoarsened starting from the coarsest one. The two nodes of the graph *G_L _*are initially placed randomly within the canvas. Then, at each recursion level, the nodes combined at the previous level are placed on the same location as the combined metanode. If the metanode consists of only one node from the previous recursion level, then the node is placed on the same location as the one representing it on the higher level. After such initial placing, a node-weighted version of the force-directed placement algorithm is applied on each recursion level. To speed-up the calculation of the force-directed placement, we made here the use of M-trees in order to quickly fetch the neighboring nodes of the node for which a new position is being calculated. The M-tree is an index structure that enables efficient indexing and querying of spatial data in metric spaces [32,33]. As another modification for biological applications, we implemented an option that allows improved separation of dense clusters according to their clustering coefficient (CC, the number of edges connecting the neighbors of the node divided by the maximum possible number of such edges). The clustering option emphasizes the attractive forces of those nodes with high CC-values towards their connected neighbors and also emphasizes the repulsive forces between the network clusters [31] (Additional File 1).

### Implementation of the algorithm

The Java implementation of the multilevel layout algorithm is distributed as an open-source Cytoscape plug-in, named Multilevel Layout, licensed with GNU GPLv2 license [34]. The plug-in can be used either with default or customized settings (Additional File 2). The most important setting is the clustering option which toggles on or off the usage of clustering coefficient in the layout calculation (by default it is on). The natural spring length setting can be used to scale up or down the resulting layout, which may be needed with very large graphs having several recursive coarsening levels. In most cases, however, the user can simply rely on the default setting, since such downscaling is done automatically during the layout calculation if the resulting layout becomes too wide. Problems with extreme wide networks may occur in the form of abnormal termination of the algorithm, if the inter-node distance underflows the decimal scale of the programming language. In addition, there are two other layout user-settings: a constant multiplier for repulsive force calculation (a higher value increases the effect of the repulsive force), and a tolerance parameter which controls the convergence of the algorithm (a higher value results in faster convergence). The user can also choose whether the original weighting function or its degree-modified version is used in the node matching process.

### Biological evaluation procedure

To facilitate biological evaluation of the layout algorithms and their solutions, we developed and implemented an additional plug-in for Cytoscape, named Biological Evaluation plug-in, for which implementation, source-code and user-instructions are freely available from website [35]. The idea behind the evaluation procedure is to compare the two-dimensional layout generated by a layout algorithm against an external biological evaluation criterion. More specifically, our procedure reviews all the connected edges of the layout in the order of their Euclidean distances, and evaluates such increasing neighbor sets with respect to a Gene Ontology (GO)-based semantic similarity of the corresponding gene products [36]. Semantic similarity has been widely used for biological evaluation of many bioinformatic approaches [37]. Our implementation constructs a GO structure in the computer memory such that it can be used to efficiently query the semantic similarity between gene or protein nodes in the layout [38].

The output of the evaluation plug-in is a graphical evaluation chart, which depicts how well the information content of the network layout agrees with the biological process ontology stored in the GO (Additional File 3). As the percentage of evaluated neighbors increases towards 100%, the average semantic similarity of each algorithm approaches the random trace, which represents the average semantic similarity of the whole network. As an upper bound, the evaluation chart includes also the theoretical optimal case, which represents the ideal situation in which the ranking of the node pair according to their layout distance equals the ranking based on the semantic similarity of these pairs in the GO. Consequently, a trace in the chart that is closest to such optimal line suggests that the evaluated layout algorithm tends to produce the most biologically meaningful layout for a given network. To account for randomness in the layout algorithm, we repeated the layout generation and evaluation multiple times, and the results shown in the evaluation chart are averages over the runs.

To summarize the evaluation traces into single a statistic for each layout algorithm, we calculated a semantic similarity score (area between the semantic similarity trace of the algorithm and the random trace divided by the area between the optimal and random traces). The higher the score, the more biologically meaningful is the layout solution, whereas values close to zero correspond to random selection of nodes. The normalized score makes it also is easy to compare the evaluation results across a number of interaction networks. The semantic similarity scores from the different layout algorithms were compared separately for the physical interaction and genetic interaction networks. The relative improvement obtained with the multilevel approach was evaluated by comparing its performance against the other layout algorithms. Statistical significance of the observed differences between the algorithms in their semantic similarity scores was assessed using the paired *t*-test, where the *p*-value is calculated by the means of the two-tailed Student's *t*-distribution. Three different significance levels were used: *p *< 0.0005, *p *< 0.005 and *p *< 0.05.

### Test network data

To evaluate the performance of the layout algorithms implemented in Cytoscape, we used 11 interaction networks of budding yeast (*Saccharomyce Ceravisiae*), representing a wide range of topological properties (Table 1). In particular, we focused on two particular types of relationships the network are typically encoding: physical links based on screening of pairwise protein-protein interactions (PPI) or multiple protein co-complex associations (CCA), and genetic interactions between pairwise gene deletions, which reflect the relative effect of a mutation in one gene on the phenotype of a mutation in another gene. It has been shown that the genetic interaction networks encode functional information that is supplemental to that obtained from the physical protein interactions or complexes [29,39,40].

**Table 1:** Interaction networks used in the study

Network name	Ref	Type	Screen	Nodes	Edges	D	MND	MND_SD_	MCC
Ito-Core	[42]	PI	PPI	426	568	0.006	2.667	3.919	0.093

VonMering	[49]	PI	PPI	573	2097	0.013	7.319	9.017	0.450

Schwikowski	[50]	PI	PPI	1297	1862	0.002	2.871	3.109	0.125

Y2H-CCSB	[43]	PI	PPI	964	1598	0.003	3.315	5.456	0.095

Y2H-Union	[43]	PI	PPI	1647	2682	0.002	3.257	5.334	0.086

AP/MS-Combined	[45]	PI	CCA	1004	8319	0.017	16.57	18.63	0.648

LC-Multiple	[48]	MT	LCI	1213	2621	0.004	4.322	4.533	0.337

Secretory-Map	[53]	GI	E-MAP	409	4175	0.050	20.42	23.82	0.251

Chromosome-Map	[54]	GI	E-MAP	735	17,185	0.064	46.76	43.61	0.233

Costanzo	[55]	GI	SGA	4319	74,984	0.007	29.96	41.86	0.062

Costanzo-Stringent	[55]	GI	SGA	3811	35,924	0.004	16.07	22.92	0.046

Network types: PI, physical interactions; GI, genetic interactions; MT, mixed type. Screening methods: PPI, protein-protein interaction screen; CCA, protein co-complex association mapping; LCI, literate-curated interactions; E-MAP, epistatic miniarray profiling; SGA, synthetic genetic array mapping. Topological parameters: D, density; MND, mean node degree; MND_SD_, standard deviation of MND; MCC, mean clustering coefficient of the network. Costanzo-Stringent sub-network was constructed using the interaction score cut-offs ε < -0.17 or ε > 0.21. In each network, we extracted the largest connected component to be used in the evaluations.

The test networks included five of the physical interaction datasets available from the CCSB Interactome Database [41]. The first one is from the early study by Ito et al. [42], who used their high-throughput mapping system, based on yeast two-hybrid (Y2H) screens, to identify pairwise two-hybrid interactions in all possible combinations between the proteins of the *S. Ceravisiae *(Ito-Core). In each network, we extracted the largest connected component to be used in the evaluations. The 'second-generation' high-quality Y2H dataset is from the recent study of Yu et al. [43], in which they carried out a proteome-scale high-throughput Y2H screen in triplicate (Y2H-CCSB). An integrated PPI dataset was also constructed by Yu et al. by combining the Y2H-CCSB and Ito-Core networks with the PPI data obtained from another Y2H screen of Uetz et al. [44] (Y2H-Union).

A rather different type of physical network was constructed by Collins et al. [45], who combined two independent screens carried out by Gavin et al. [46] and Krogan et al. [47], in which CCA links were identified on a large-scale using affinity-purification followed by mass spectrometry (AP/MS-Combined). The Y2H and AP/MS data are of complementary nature resulting in PPI networks with different topological and biological properties. In particular, the CCA networks emphasize the complex membership, resulting in higher clustering coefficient (Table 1). The fifth dataset from the CCSB Interactome Database consists of both physical and genetic interactions, constructed trough literature-curative analysis of online publications initially by Reguly et al. [48], and further refined and filtered later by Yu et al. [43], such that interactions were curated from two or more publications (LC-Multiple).

The two other physical interaction datasets were downloaded from two published PPI network analyses. In the first one, von Mering et al. [49] combined the PPI data from the previous studies by Ito et al. [42] and Uetz et al. [44], with the aim of using the combined interaction dataset as a reference network when comparing different Y2H screening approaches for discovering physical protein interactions in yeast (VonMering). In the second study, Schwikowski et al. [50] analyzed yeast physical interactions available from public databases, such as the yeast proteome database and the MIPS database, and from previous large-scale studies, such as those of Ito et al. [42] and Uetz et al. [44]. However, they included only direct interactions discovered trough biochemical binding experiments or Y2H screening, thus leaving out those protein complexes for which the protein contacts were unknown (Schwikowski).

The four genetic interaction networks used in the evaluations came from two technically different quantitative interaction screening approaches; epistatic mini array profiling (E-MAP) from the Krogan Lab Interactome Database [51], and synthetic genetic array (SGA) mappings from the Boone Lab DRYGIN Database [52]. Two E-MAP datasets were used here, in which the mapping approach was applied to the genes involved either in the yeast early secretory pathway (Secretory-Map) [53] or in various aspects of chromosome biology (Chromosome-Map) [54]. In contrast to these selected sets of pairwise deletion mutants in yeast, Costanzo et al. [55] constructed an unbiased genetic interaction screen by applying their SGA approach on whole-genome scale. We used this high-dimensional dataset subject to two interaction scoring cut-offs (Costanzo and Costanzo-Stringent; Table 1).

## Results

The performance of the Multilevel Layout algorithm, with and without the clustering option (referred to as MLL and MLL-C), was compared against three built-in layout algorithms in Cytoscape. Force-directed layout (FDL) is a variant of the widely used node placement algorithm by Fruchterman and Reingold [23], thus representing a baseline reference for the MLL. The Cytoscape implementation takes an advantage of the efficient force-calculation algorithm by Barnes and Hut [56]. Cytoscape's Spring-embedded layout (SEL) implements a variant of the layout algorithm introduced by Kamada and Kawai [22]. The algorithm is based on the idea of minimizing the total energy of the network by calculating partial differential equations of the energy function and moving the nodes accordingly. The FDL and SEL algorithms represent popular open-source solutions, capable of producing visually pleasant layout solutions, especially for relatively small and simple network structures [6,10]. The yFiles Organic layout (ORL) is a proprietary closed-source implementation of the force-directed placement paradigm, which combines elements from several layout algorithms to facilitate identification of clusters of tightly connected network modules [20], hence sharing the same objective with the MLL-C.

### Run-to-run variability of the layout solutions

The evaluation runs were performed on a laptop with Intel i7 Q740 processor and 6 GB RAM running Windows 7 OS. The four layout algorithms were run using their default parameter settings in Cytoscape version 2.8.1 [57]. The layouts of the ORL algorithm remain relatively constant from run-to-run, except for smaller-scale networks, and therefore its evaluation was repeated 5 times. The rest of the layout algorithms are more non-deterministic, resulting in some degree of between-run variability, and therefore their performance was assessed based on several runs: 10-20 initializations for MLL and FDL and a maximum of 10 repeats for the SEL because of its relatively high computational complexity. The results were averaged over the replicate runs and the variability in computational times and semantic similarity scores was assessed with standard error of the mean (SEM). In general, ORL and FDL showed lowest variability over the replicate runs, followed by MLL, MLL-C and SEL (Additional File 4). Although the MLL-C algorithm is sensitive to the random initialization, its layout solutions preserve the main characteristics of the underlying network topology, such as highly connected hub nodes or network clusters, even if these may end up being in different locations in the different runs (Additional File 5). Therefore, the MLL-C algorithm is capable of producing topologically consistent layout solutions, the biological relevance of which is evaluated in the next sections.

### Semantic similarity in the physical networks

**Figure 2:**
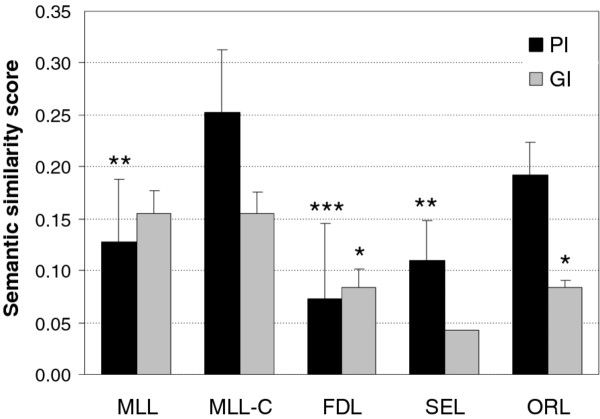
**Biological evaluation of the layout algorithms**. The black and grey bars show the average semantic similarity over the networks of physical interactions (PI) and genetic interactions (GI), respectively. Error bars show the standard error of the mean (SEM). Only one of the GI networks could be laid out using the SEL algorithm in less than one hour. The performance of the other algorithms was compared against the multilevel layout with the clustering option on (MLL-C): *** *p *< 0.0005; ** *p *< 0.005; * *p *< 0.05, two-sided paired *t*-test.

**Figure 3:**
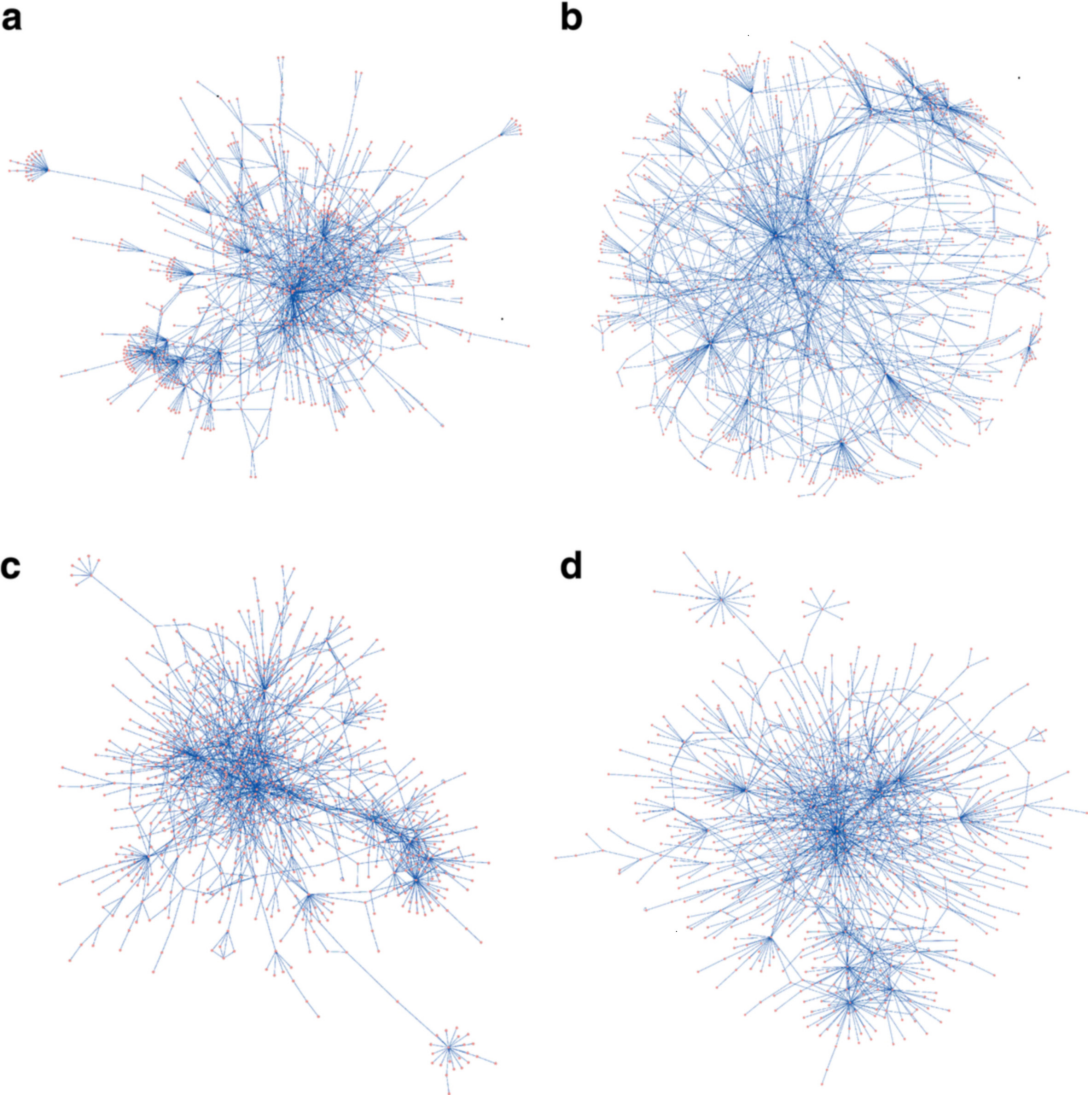
**Example layouts for the CCSB-Y2H network**. **(A) **yFiles Organic layout (ORL). (**B**) Cytoscape's Spring-embedded layout (SEL). (**C**) Cytoscape's Force-directed layout (FDL). (**D**) Multilevel layout with the clustering option (MLL-C). The number of nodes and edges in the CCSB-Y2H network are 964 and 1598, respectively, with an average clustering coefficient of 0.095. The node sizes and edge widths were standardized in Cytoscape to make the layout displays comparable in accuracy (the same zooming resolution was used in the export).

Compared to the popular layout algorithms in Cytoscape platform, the layout solutions produced by the versions of the MLL algorithm captured relatively well the underlying biological processes of the various test networks (Figure 2). In particular, when using the clustering option of the algorithm, the biological information contents of the MLL-C layout solutions were significantly higher than those of the FDL or SEL solutions in the physical test networks (*p *< 0.0005 and *p *< 0.005, respectively, paired *t*-test). The yFiles ORL algorithm obtained the best semantic similarity score in three of the 11 test networks; these networks encode Y2H protein-protein interactions, co-complex membership associations and literature-curated physical and genetic interactions (Additional File 4). Among these three networks, the AP/MS-Combined protein complex network represents a rather unusual case with exceptionally high clustering coefficient (Table 1). In the two other networks, the semantic similarity scores are close to each other with MLL-C and ORL. In fact, in the Y2H-CCSB network, MLL-C showed best semantic similarity among the nearest network neighbors, whereas the ORL layout outperforms the others when going to more distant node pairs (Additional File 3). However, both MLL-C and ORL generated visually balanced network layouts, with marked cluster structures, whereas SEL and FDL resulted in more ball-like or prolonged solutions (Figure 3).

### Semantic similarity in the genetic networks

To test whether the good performance of the ORL algorithm in the mixed physical and genetic interaction network will also hold when analyzing purely functional relationships, we evaluated its relative performance on four large-scale genetic interaction networks (Table 1). The evaluation results changed quite dramatically when focusing on these functional interaction networks. In general, the semantic similarity scores were at a lower level compared to the physical protein interaction networks (Figure 2). When comparing the different layout options, the MLL-C algorithm showed increased biological information content beyond those of the FDL, SEL or even ORL (*p *< 0.05, paired *t*-test). Interestingly, the performance of the FDL was similar both in the physical and genetic interaction networks. Moreover, it seems that the clustering option of the multilevel algorithm is not necessary when drawing genetic interaction networks. In fact, the MLL algorithm without using the clustering option provided even slightly better results in the smaller one of the two E-MAP networks (Secretory-Map; Additional File 4). However, the differences between the two MLL modes were relatively small compared to the performance of the other layout options on the genetic interaction networks (Additional File 3). Besides the network type, no other network parameter could explain the variation in the semantic similarities across the layout solutions either in the physical or genetic interaction networks (Additional File 6).

### Running time of the algorithms

**Figure 4:**
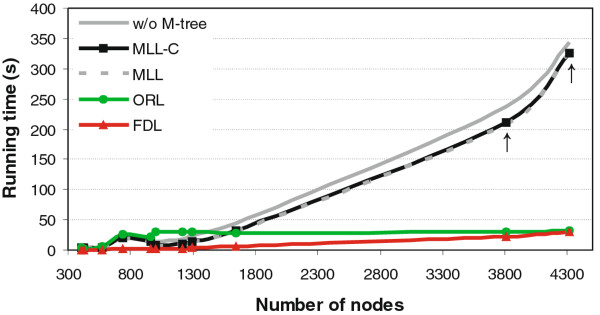
**Computation times of the layout algorithms**. The running times (in seconds) are plotted as a function of the network size (number of nodes). The number of the nodes correlated significantly with the running times of the MLL, MLL-C (with or without the M-tree option) and FDL algorithms (*p *< 10^-5^). The running time of the proprietary ORL implementation converged after 1000 nodes. The systematically slowest SEL algorithm was omitted from the illustration, since it could draw only 7 out of the 11 test networks in less than one hour. The arrows point the two largest SGA genetic interaction networks (Costanzo and Costanzo-Stringent).

Among the layout algorithms tested in Cytoscape, FDL was systematically the fastest and SEL systemically the slowest layout option (Figure 4). As expected, the number of nodes and edges in the network were the most predictive network properties for the computation time of most layout algorithms, including MLL, MLL-C and FDL (*p *< 10^-5^, Pearson's correlation, *t*-distribution); however, the running time of the SEL algorithm was correlated more strongly with other parameters, such as network density, average node density or its standard deviation (Additional File 6). Notably, the SEL algorithm could draw only 7 out of the 11 test networks in less than one hour. The running time of the proprietary, closed-source ORL implementation seems to level off after 1000 nodes, regardless of the increased network complexity (Figure 4). The two MLL implementations were relatively fast on networks with less than 2000 nodes (running time less than one minute); however, for the two largest genetic interaction networks, comprising of 3811 and 4319 nodes and 35,924 and 74,984 edges, respectively, the running time of the MLL-C grows exponentially, approaching almost 4 and 7 minutes. Therefore, even if the M-tree architecture could decrease the computation times of the MLL-C algorithm, especially in larger and moderately dense networks (Additional File 7), further speed-up would be advantageous, especially when moving towards extremely large and densely connected networks.

## Discussion

We have implemented a multilevel network layout algorithm and shown that it can generate visually pleasant and biologically meaningful layouts for a wide spectrum of biological network structures. In general, the Multilevel Layout (MLL) plug-in provided layout solutions and network views that are complementary to those of the built-in layout options of Cytoscape; it demonstrated an added value especially in large-scale networks representing either pairwise functional or physical interactions between genes or proteins.

A particular network type in which the multilevel algorithm showed an improved performance involved the complex networks of genetic interactions. In contrast to the physical PPI networks, emphasizing densely interconnected network clusters in the functional genetic interaction layouts did not seem to increase the information on the biological processes, as was demonstrated by the reduced semantic similarity of ORL, and also of MLL-C to some extent, compared to the MLL without the clustering option. This indicates that genetic interaction modules encode also a wide range of functional cross-talk across multiple biological pathways. Such quantitative genetic networks are increasingly being mapped in model organisms to study many fundamental questions, such as genotype-phenotype relationships and buffering of genetic variation [25,28,58]. Genetic interactions are also involved in many human disease phenotypes, such as cancers and cardiovascular diseases. As an example, a statistical epistasis network was recently constructed based on SNP-data to study the global architecture of gene-gene interactions, as well to identify higher-level relationships and inter-connected modules involved in bladder cancer [59]. Application of the multilevel layout algorithm to such emerging networks should prove useful for many network biology and network medicine applications.

**Figure 5:**
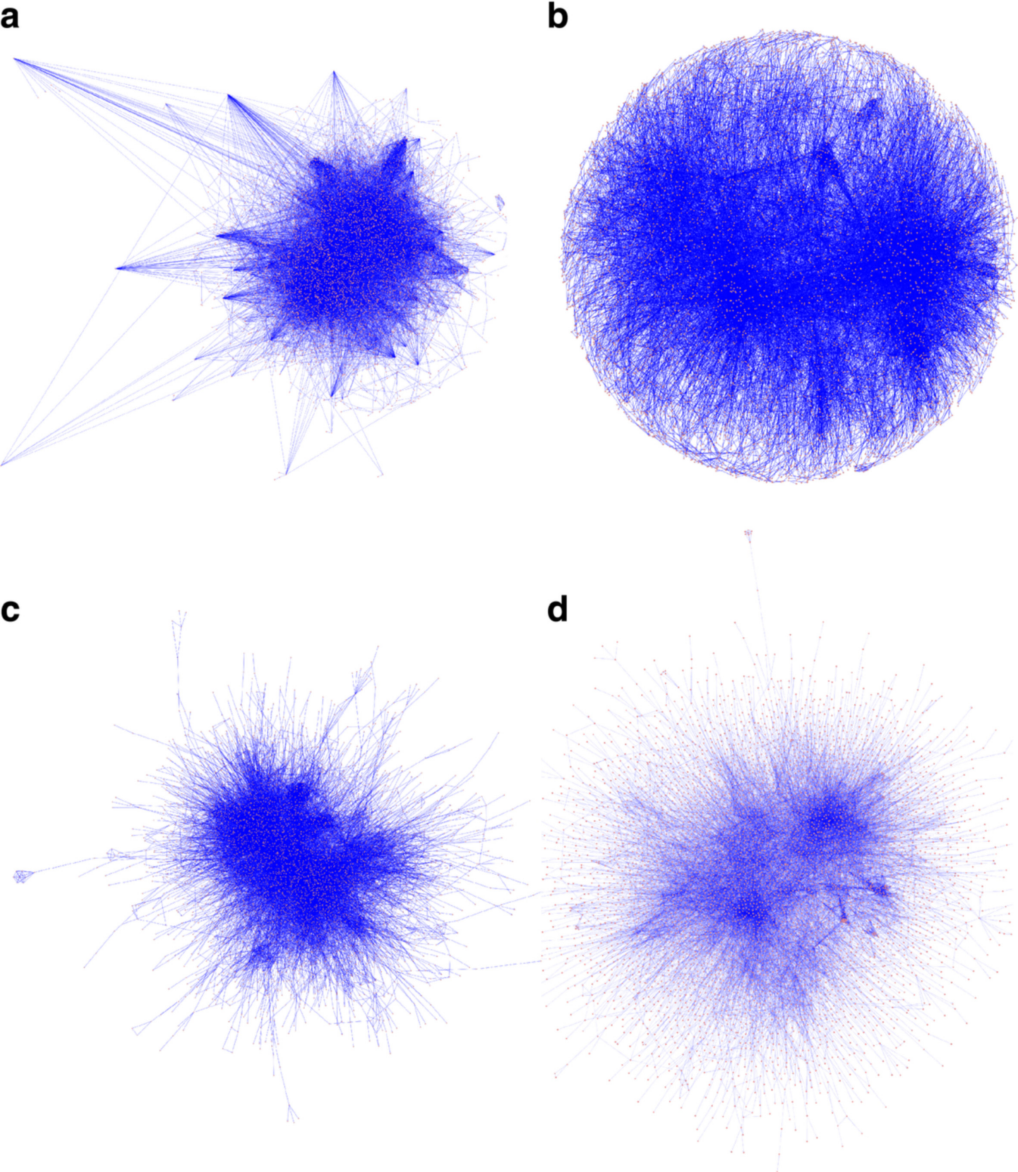
**Example layouts for the HPRD network**. **(A) **yFiles Organic layout (ORL). (**B**) Cytoscape's Spring-embedded layout (SEL). (**C**) Cytoscape's Force-directed layout (FDL). (**D**) Multilevel layout with the clustering option (MLL-C). The HPRD network consists of 5699 protein nodes and 19,779 literature-curated protein-protein interactions. A high-resolution version of the layout solutions of the four layout algorithms is provided in Additional File 8.

In addition to the multilevel layout, we have also introduced here a novel way of evaluating layout solutions in terms of their biological relevance. The implementation of the Biological Evaluation plug-in enables one to evaluate layout solutions from any existing or future graph drawing algorithm and to optimize its performance for a given network under the analysis. In the present work, the comparative evaluations were carried out on yeast interaction networks, since the GO annotations in *S. Ceravisiae *are relative accurate and established, compared to many other organisms. Beyond the systematic evaluations presented here, we have been extensively testing and applying the MLL plug-in also in other organisms. For instance, when applied to the literature-curated Human Protein Reference Database (HPRD) network [60], the multilevel organization was able to visualize both the global and local network structures, such as the large number of peripheral protein nodes and highly interconnected sub-network modules, which were largely missed by the other layout algorithms in Cytoscape (Figure 5; Additional File 8).

After generating the layout, the resulting networks can be investigated in more detail, for example, by zooming into densely-connected clusters. There is a wide range of clustering algorithms introduced for finding such sub-network modules [61], some of which are also available in network analysis software, including MCODE [62] or Graphle [63]. As an example, we searched here for the top-scoring clusters in the Schwikowski network using the MLL-C layout and ClusterViz Cytoscape plug-ins (Additional File 9). To facilitate revealing the full spectrum of hierarchical modularity of a network, one could also incorporate a cluster detection phase explicitly within the multilevel framework, hence providing a multi-resolution viewing of the modules as communities or metanodes, similarly as was done in the GenePro [64] or GLay [65]. Combining all of the nodes in the detected clusters could also further improve the multilevel layout solutions, especially in networks such as the AP/MS-Combined, which show exceptionally high and extensive clustering structure. The good performance of the organic layout in this network indicates that there is still potential for further improvement.

Instead of first detecting sub-network clusters or modules based on the network connectivity and then contrasting these against known complexes or pathways, an alternative approach is to use such external biological information directly to optimize the placement of the nodes or multinodes [12,66-71]. Here, we chose not to use any additional information in guiding the layout process, since this could bias the biological evaluation of the layout algorithms, and because reliable external data may not be always available, for instance, when studying human interaction networks. However, one could extend the multilevel layout framework to incorporate also additional information, such as user-defined GO annotations or other node attributes, in the node matching phase, in order to better emphasize biologically meaningful aspects of the network topology. The multilevel framework can also be combined with other algorithms than the force-directed layout in order to improve or modify the final layout result. The yFiles organic layout would be an interesting option to include; however, its proprietary implementation is not publicly available.

A limitation of the current implementation of the multilevel layout algorithm is its relatively lengthy running times in the largest network graphs. For instance, generating the layout took almost 7 minutes for the largest yeast genetic interaction network (4319 nodes with 74,984 edges) and 8 minutes for the human HPRD-PPI network (5699 nodes with 19,779 edges). While the M-tree architecture resulted in somewhat reduced computation times, further speed-ups could be achieved by linking specific C functions to the Java implementation [65], or by using hardware-based graphics acceleration in Cytoscape [72]. For instance, performance benefits could be obtained through full usage of the power of graphics processing units. Additional decrease in the layout calculation times will likely be obtained by making better use of multiple cores in the future versions of Cytoscape. For example, the Intel i7 processor could handle eight simultaneous processing threads, making it suitable for parallelized layout calculation once the Cytoscape platform is capable of supporting multi-threading and effective parallelization.

## Abbreviations

AP/MS: Affinity-purification/mass spectrometry; CC: Clustering coefficient; CCA: Co-complex association; FDL: Force-directed layout; GO: Gene ontology; E-MAP: Epistatic miniarray profiling; HPRD: Human protein reference database; MLL: Multilevel layout; ORL: Organic layout; PPI: Protein-protein interaction; SEL: Spring-embedded layout; SGA: Synthetic genetic array; Y2H: Yeast two-hybrid.

## Competing interests

The authors declare that they have no competing interests.

## Authors' contributions

TA conceived the study and ON participated in its design. PS, JT and HV implemented the Multilevel Layout plug-in. JT developed and implemented the Biological Evaluation plug-in. JT analyzed the datasets. JT, PS, ON and TA wrote the manuscript. All authors read and approved the final manuscript.

## Supplementary Material

Additional file 1 **Internal TUCS Technical Report by Salmela et al. (2008)**. Click here for file

Additional file 2 **User-adjustable settings for the Multilevel Layout plug-in**. Click here for file

Additional file 3 **Comparison of the layout algorithms using semantic similarity**. Click here for file

Additional file 4 **Running times and semantic similarities for the test networks**. Click here for file

Additional file 5 **Multiple runs of the MLL-C algorithm in the Ito-Core network**. Click here for file

Additional file 6 **Correlation coefficients for running times and semantic scores**. Click here for file

Additional file 7 **Relative speed-up of MLL-C provided by the M-tree architecture**. Click here for file

Additional file 8 **Layout solutions when applied to the human HPRD network**. Click here for file

Additional file 9 **The top-scoring clusters found in the Schwikowski network**. Click here for file
