# Supplementary material for: A multilevel layout algorithm for visualizing physical and genetic interaction networks, with emphasis on their modular organization
Source: BioData Min. 2012 Mar 26;5:2. doi: 10.1186/1756-0381-5-2 (PMC3342218; doi:10.1186/1756-0381-5-2)
Supplement: Additional file 1 — Internal TUCS Technical Report by Salmela et al. (2008). [file 1756-0381-5-2-S1.PDF]

TUCS

Pekka Salmela | Olli S. Nevalainen | Tero Aittokallio

A Multilevel Graph Layout Algorithm for Cytoscape Bioinformatics Software Platform

Turku Centre *for* Computer Science

TUCS Technical Report
No 861, January 2008

A Multilevel Graph Layout Algorithm for Cytoscape Bioinformatics Software Platform

Pekka Salmela
University of Turku, Department of Information Technology and TUCS

Olli S.Nevalainen
University of Turku, Department of Information Technology and TUCS

Tero Aittokallio
University of Turku, Department of Mathematics and TUCS

TUCS Technical Report
No 861, January 2008

Abstract

We have implemented a graph layout algorithm of C. Walshaw as a plug-in in the Cytoscape bioinformatics software platform. The algorithm combines traditional force-directed placement of graphs with an efficient multilevel framework in order to generate clear and aesthetic layouts. It is targeted especially at displaying large graphs with several thousands of nodes. Our plug-in, named Multilevel Layout Plugin (or MLLP) works by generating a set of increasingly coarser graphs and applies force-directed placement to each level and advances gradually towards final layout. The algorithm works in reasonable time and is robust for different kinds of graphs to be drawn.

Availability: MLLP is released under GNU General Public License and is available at http://chianti.ucsd.edu/cyto_web/plugins/

Keywords: graph layout, multilevel framework, Cytoscape

TUCS Laboratory
Algorithmics Laboratory, Biomathematics Laboratory

1. Introduction

A common way to describe dependencies in data sets is to model them as graphs, where nodes stand for specified data objects and edges express connections between them. This method is appropriate especially for biological data, such as modeling gene regulation networks and protein interactions. In order to visualize a graph so that its essential properties are clearly highlighted, a way to lay out the graph structure in two dimensions is needed. A number of different layout algorithms have been proposed and implemented in different data analysis software. One such a software package is Cytoscape (Shannon et al., 2003), which is an open source bioinformatics software platform for visualizing molecular interaction networks and integrating these with various state information. At the moment, layout algorithms in Cytoscape are mostly based on a type of force-directed placement such as those presented by Fruchterman and Rheingold (1991) or Kamada and Kawai (1989). While these algorithms perform quite well with graphs with less than thousand nodes, more efficient algorithms are needed for large-scale experimental data sets containing thousands of nodes. To this end, we present Multilevel Layout Plug-in (MLLP), a layout plug-in for Cytoscape, which implements a multilevel framework combined with a modified version of the classical force-directed placement method. The original version of the multilevel force-directed algorithm was introduced by C. Walshaw (2003), and in order to cut down the running time we have made some modifications to the algorithm and implemented it in Cytoscape.

2. Method

The multilevel framework combined with the force-directed placement for determining graph layouts achieves its result in two separate phases. The first part of the algorithm generates a set of increasingly coarser graphs, G_0, G_1, ... , G_L, where G_0 is the original graph for which the layout is calculated and G_L is the coarsest graph consisting of only two nodes and one edge between them. The graph G_i is said to be on the level i of the graph hierarchy, or to be the level i of the progress of the multilevel algorithm.

The graphs are coarsened by finding a maximal independent subset of edges and by pairwisely combining the nodes connected by these edges to form nodes in the graph on the next level. If there are nodes without suitable matching pairs in G_{i-1}, those nodes form a node in the graph on the next level G_i by themselves. A set of edges is independent if no two edges in the set are incident on the same node, and maximal if it is not possible to construct a bigger set without breaking the independence criterion. There are methods to solve the maximal independent set problem optimally (Papadimitriou & Steiglitz 1982), but as the speed of the algorithm is here crucial, a non-optimal method is used.

The original method proposed by Walshaw was to pick a random node and match it with a neighboring node with the smallest weight. For the graph G_0 the weight of a node is

defined to be 1 for each node. Since weighted nodes in the coarsened graphs represent sets of nodes from the original graph G_0, the weight of a coarsened node is equivalent to the number of original nodes in the set it represents. If more than one node share the same weight, then the matched node is chosen randomly. The method is fast to perform, but it may cause problems with certain kinds of graphs, especially with those with many "star-like" formations. For example, let us consider finding a maximal independent subset of edges using the method described above in the graph presented in the Figure 1. If the first randomly chosen node is A, and the randomly chosen neighbor of A is D, the consequence is that neither of the nodes C or B can be matched with their neighbors. Furthermore, if the chosen neighbor of node A is node H, none of the nodes I, J, K or L can no more be matched with any of their neighboring nodes. Thus the size of the maximal independent subset will be small and the graph is coarsened only a little. According to this simple example, it seems that choosing a neighbor with a high degree (number of edges incident to the node) decreases the number of nodes available for matching.

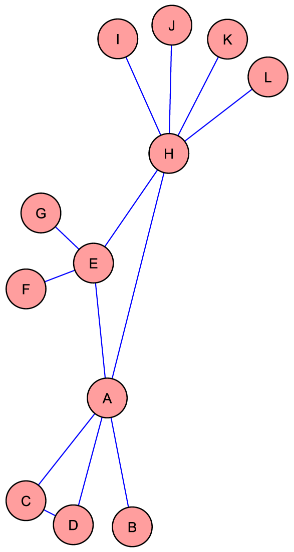

Figure 1: A graph with "star-like" formations.

To avoid this problem we present an alternative rule, in which we choose the neighboring node *n* to be matched such that the function *f(n) = n.weight + n.degree* will be minimized. If there are several such nodes, the node *n* is chosen randomly amongst them. Function *f(n)* combines the original idea of favoring nodes with small weight with our proposal to favor nodes with low degree. For coarse graphs containing small number of high weighted nodes with relatively low degrees, the term *n.weight* dominates the result of the function, and thus the result focuses on balancing the node weights evenly. On the other hand, when dealing with more detailed graphs containing low weighted nodes with higher degrees the dominating term is *n.degree* and as stated above, the number of possible matching pairs of nodes is increased. When testing with Cytoscape the revised node selection rule performed substantially faster than the original one with no obvious negative impact on the resulting layout.

In the second phase of the multilevel framework, the graphs G_L, G_{L-1}, ... , G_1 are uncoarsened starting from the coarsest one. Firstly, the two nodes of the graph G_L are placed randomly within the canvas. Then, for each recursion level the two nodes combined at the previous level are placed at the same location as the combined node. If no matching pair was found for a node, that node is placed at the same location as the one representing it on the higher level. This is done for all the combined pairs. After this, a node-weighted version of the force-directed placement algorithm is applied on that recursion level. For a graph $G = (V, E)$ the algorithm works as follows (Walshaw 2003):

```
FUNCTION forceDirectedPlacement(){
  WHILE(converged ≠ TRUE){
    converged = TRUE
    FOR(v ∈ V){
      Θ = 0
      FOR(u ∈ V, u ≠ v){
        Δ = u.position − v.position
        Θ = Θ + (Δ/|Δ|)·f_r(|Δ|, u.weight)
      }
      FOR(u ∈ v.neighborhood){
        Δ = u.position − v.position
        Θ = Θ + (Δ/|Δ|)·f_a(|Δ|)
      }
      newPosition = v.position + (Θ/|Θ|)·min(t, |Θ|)
      Δ = newPosition − v.position
      IF(|Δ| > k·tolerance) converged = FALSE
      v.position = newPosition
    }
    t = cool(t)
  }
}
```

Here k is the ideal spring length (see below), *tolerance* is a parameter defaulting to 0.01 and *cool*(t) is a function returning the value $\lambda \cdot t$, where λ is a parameter defaulting to 0.9. The repulsive and attractive forces f_r and f_a are calculated according to the two following formulae:

$$f_r(x, w) = -Cwk_i^2/x$$
$$f_a(x) = x^2/k_i,$$

where C is a constant multiplier (defaulting to 0.2) and k_i is the ideal spring length for the level i, calculated as follows

$$k_i = \begin{cases} \sqrt{\frac{4}{7}} \cdot k_{i+1} & \text{, if } 0 \le i < L \\ \frac{1}{|E_L|} \sum_{(u,v) \in E_L} |(u,v)| & \text{, if } i = L \end{cases}$$

As G_L consists of only two nodes and one edge, the ideal spring length for level L is simply defined as the distance between the two nodes.

In the MLLP we have also implemented a user option that allows to emphasize dense clusters according to the graph topology. This option uses the information about clustering coefficient values (CC-values) of the nodes and emphasizes the attractive forces of the nodes with high CC-values towards their connected neighbors. The clustering coefficient C_i of a node v_i is the proportion of the number of existing edges between neighborhood nodes of v_i divided by the number of edges that could possibly exist between them. The neighborhood N_i of the node v_i is defined as

$$N_i = \{v_j \in V | e_{ij} \in E\}$$

Here e_{ij} denotes an edge between nodes v_i and v_j. Thus, the clustering coefficient C_i for v_i is defined as follows:

$$C_i = \frac{2|\{e_{jk} \in E | v_j, v_k \in N_i\}|}{|N_i|(|N_i|-1)} : v_j, v_k \in N_i, e_{ij} \in E$$

At the beginning of the calculation of the force-directed placement, the CC-values are calculated for each node and stored in a hash table for a fast access. The repulsive and attractive forces affecting the node v are then modified as follows:

$$f_r = \begin{cases} f_r & \text{, if } C_u < T \\ \dfrac{f_r}{(1 + C_u \cdot M)} & \text{, if } C_u \geq T \end{cases}$$

$$f_a = \begin{cases} f_a & \text{, if } C_u < T \\ f_a \cdot (1 + C_u \cdot M) & \text{, if } C_u \geq T \end{cases}$$

Here, *u.CC* is the clustering coefficient value of node *u*, *T* is a threshold value (defaulting to 0.3) below which the clustering coefficient has no effect, and *M* is a constant multiplier (defaulting to 3.0).

3.Test results

A variety of tests were performed with the MLLP. In particular, we wanted to find out how well the MLLP performs against the built-in layout methods in Cytoscape, to evaluate the effect of the new node selection rule on its running time and to demonstrate the emphasation of the dense clusters by the use of CC-values. The size of the graphs used in testing ranged from relatively small ones containing 178 nodes and 464 edges to large ones including 5699 nodes and 19779 edges. The tests were performed on a desktop computer with Intel Pentium D 840 processor (dual core, 3.2 GHz each) and 2048 MB RAM running Microsoft Windows XP Professional operating system and using the 2.4.1 version of Cytoscape.

The two reference layout methods tested are Cytoscape Spring Embedded -layout and yFiles Organic -layout which both are widely used and are available in the normal installation of Cytoscape. Cytoscape Spring Embedded -layout is an open source implementation of an algorithm presented by Kamada and Kawai (1989). The algorithm

is based on the idea of minimizing the total energy of the system by calculating partial differential equations of the energy function and moving the nodes accordingly. yFiles Organic -layout is a proprietary force-directed placement variant combining elements from several different layout algorithms. The version in Cytoscape has a limited 30 second calculation time, which made it incomparable in terms of the computation times of the layout methods when dealing with very large graphs, but it was chosen for testing because of its good performance with smaller graphs.

The networks used in testing were:

- BINF1335_n23_r0.72: Human gene coexpression network for 897 genes over *n*=23 conditions measured on DNA microarrays. Two genes were connected if the Pearson correlation between their expression patterns was greater or equal to 0.72. The same data set was used in Elo *et al.* (2007).
- BINF1335_n23_r0.85: The same data as above with correlation threshold at least 0.85.
- AllDetected_642: Extracted from a human PPI-network by including selected proteins only.
- MCL18_m2_178: Human protein-protein interaction (PPI) data were taken from BIND, HPRD, IntAct, Reactome, BioGrid and DIP databases and clustered using the Markov clustering (MCL) with inflation parameter=1.8 (see Brohée & van Helden (2006)). This network represents a cluster (module) of 178 proteins.
- MCL18_m1_207: Another cluster from the same PPI data as above containing 207 proteins.
- DE123n1_1406: Extracted from a human PPI-network by including selected proteins only.
- ptacek: See Ptacek et al. (2005)
- rual: One of the first large scale human PPI-mappings.
- HPRD: A large scale human PPI-network predicted according to literature.

The networks were characterized by a number of attributes, including the number of nodes, the number of edges, density ($|E|/|V|$), the mean of node degrees and the clustering coefficient (Table 1).

Table 1: Test graph characteristics.

Graph name	AllDetected_642	BINF1335_n23_r0.72	BINF1335_n23_r0.85	DE123n1_1406	HPRD	MCL18_m2_178	rual
Number of nodes	642	884	757	1406	5699	178	2784
Number of edges	5341	26078	6755	19250	19779	464	6438
Density	8,32	29,5	8,92	13,69	3,47	2,61	2,31
Mean of node degrees	15,72	59	17,85	26,58	6,76	4,88	4,32
Nodes with degree = 1	139	14	79	107	1320	62	950
Nodes with degree <= 5	380	52	253	515	3745	135	2236
Nodes with degree <= 10	466	97	384	785	4695	157	2555
Nodes with degree <= 50	555	455	698	1167	5639	177	2775
Maximum node degree	100	188	85	168	160	116	129
Clustering coefficient	0,36	0,56	0,45	0,33	0,19	0,23	0,07

The quality of the layouts was evaluated by five criteria listed in Table 2. The first four have been used for example by Fruchterman and Rheingold (1991), while the last one is a simplification of an criterion used by Purchase (2001).

Table 2: The aesthetics criteria used in testing.

Criterion	Explanation
Computation time in seconds	How long it took the layout algorithm to create the layout.
Deviation of nodes along the x-axis/y-axis (scaled between [0, 100])	How evenly the nodes are spread within the canvas.
Standard deviation of the edge lengths (scaled between [0, 100])	How uniformly the edge lengths are divided.
Number of edge intersections (sample)	How many edge intersections there are among the sampled edges.
Mean of minimum of angles between adjacent edges (sample)	What is the mean of the smallest angles between adjacent edges originating from the same node.

In the above criteria a sample means that only a random subset of the nodes or edges was considered when calculating the value of the criterion. It is important to notice, that these criteria measure only some possible features that have been used in characterizing the aesthetics of graph layouts and subjective preferences of different viewers may differ greatly. It is possible though, that they do not always go hand in hand with the subjective estimate of aesthetics by the viewer.

A number of observations were made during the testing. First, it became obvious, that the MLLP is not able to compete with the two other algorithms when it becomes to pure speed of the layout calculation. This was not really a surprise, as the multilevel framework consists of several levels of hierarchy and the force-directed placement is applied on each of these levels separately. This obviously increases the running time. It might be, that the other two built-in methods have been optimized carefully and they use the functions and resources offered by Cytoscape API in a more efficient way. In this respect the code of the MLLP still needs polishing.

Secondly it was observed, that when dealing with graphs with over 2000 nodes both Y-files Organic and Cytoscape Spring Embedded -layout started to generate increasingly unwanted results. Due to the limited time for calculation, Y-files Organic -layout generated some very distorted layouts with some of the nodes "thrown" outside the main cluster in a very unaesthetic way and without any obvious reason. The Spring Embedded -layout on the other hand generated regular "ball-like" formations without any significant sign of clusters or other visual hints of the true topology of the graphs. In contrast, the MLLP generated layouts even for large graphs with no such problems, although it was rather hard to tell whether or not the images represented the true topology of the graphs. This was partly because there was no available "nice" reference image of the graphs or any information if there were clusters or other significant visual formations present in the graphs. For all the layouts produced during testing see Appendix 1.

In overall, the three layout methods performed quite evenly, although they never produced similarly looking layouts. In many occasions MLLP managed to generate layouts with less edge intersections, but it achieved its results taking more time than the Cytoscape Spring Embedded layout and, of course, the time-limited yFiles Organic layout. The benefits of MLLP in terms of the quality of the layouts produced became more visible when considering graphs with thousands of nodes. Examples of images produced by different layouts can be found in Figures 2.1-2.3. For detailed results see Appendix 2.

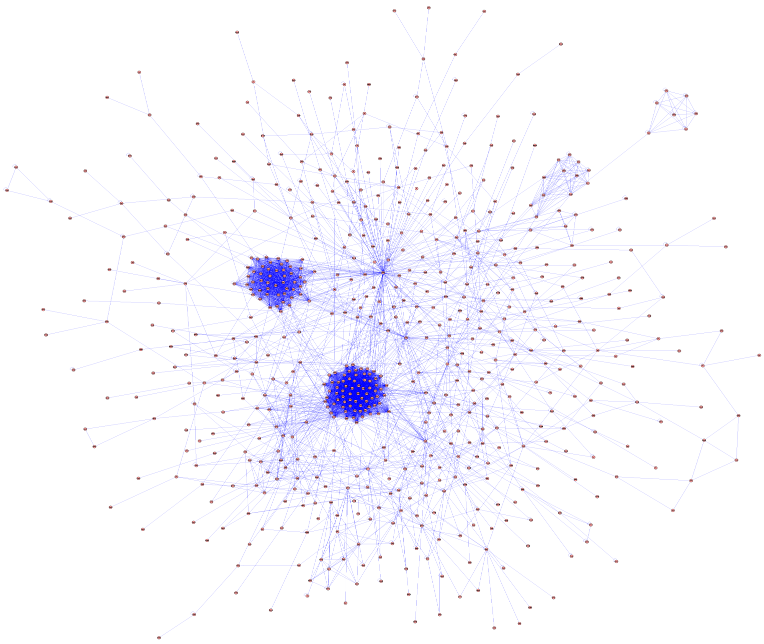

Figure 2.1: AllDetected_642 drawn by MLLP.

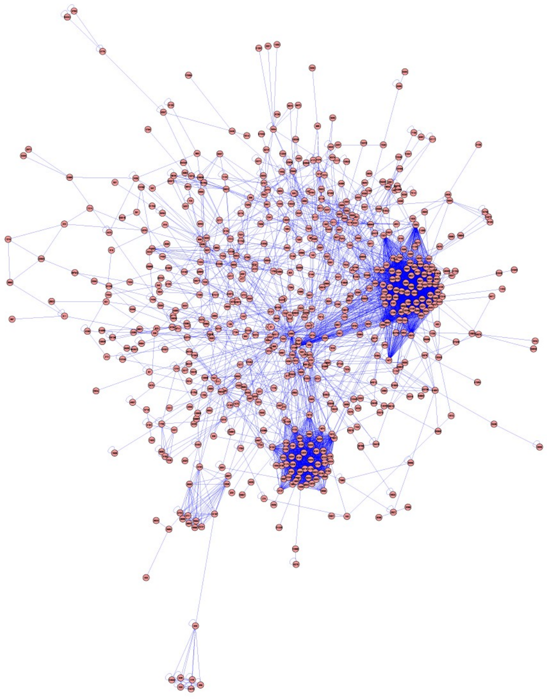

Figure 2.2: AllDetected_642 drawn by yFiles Organic layout.

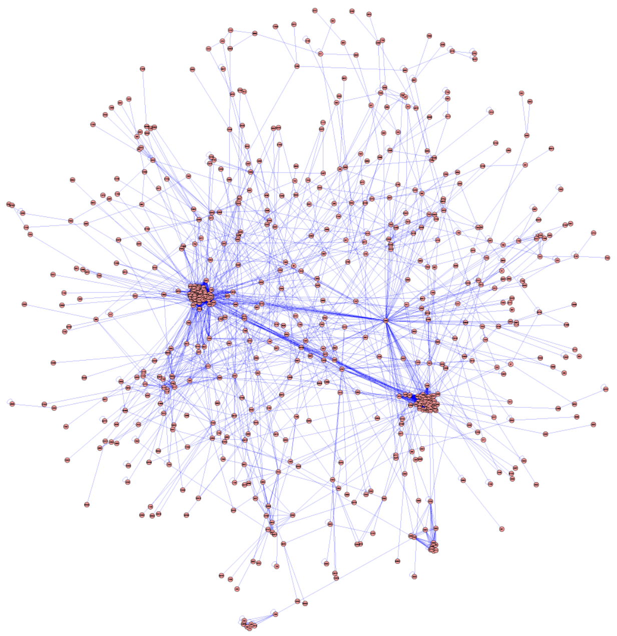

Figure 2.3: AllDetected_642 drawn by Cytoscape Spring Embedded Layout.

When testing the MLLP with and without the clustering option it became clear, that the option to draw together nodes with high clustering coefficient values was indeed able to produce more tightly drawn formations. However, due to lack of very clustered testing graphs it remained uncertain if the option is truly able to bring out clusters, that are more difficult to distinguish using the MLLP without the clustering option. On the other hand, it is clear that calculating the clustering coefficients takes some extra time; computation time increased about 10% when clustering option was turned on.

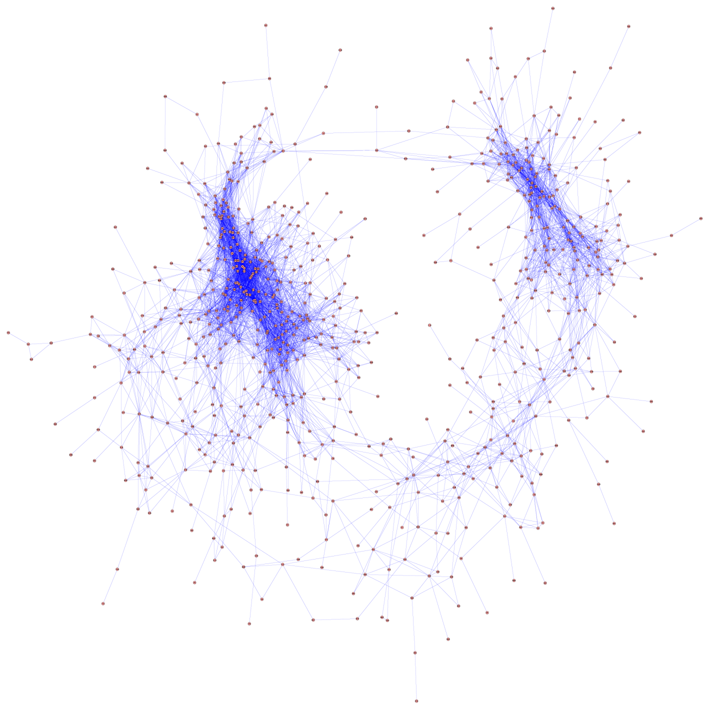

Figure 3.1: BINF1335_n23_r0.85 drawn by the MLLP when the clustering option is turned on.

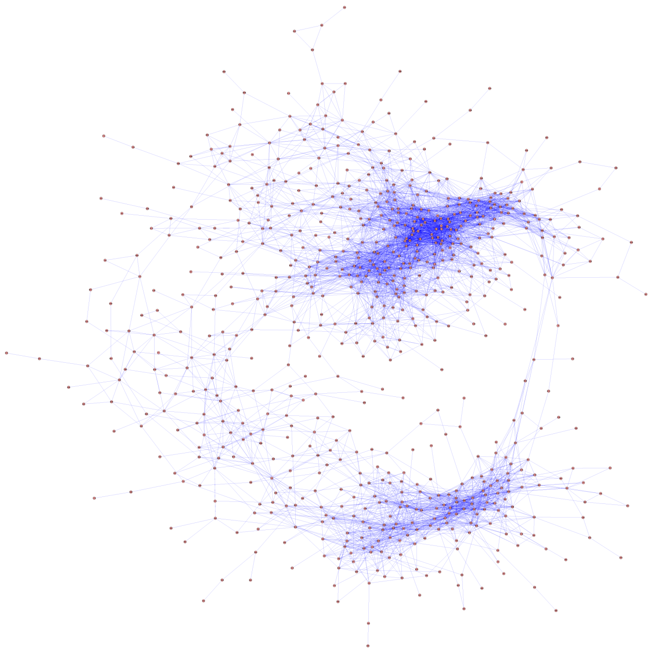

Figure 3.2: BINF1335_n23_r0.85 drawn by the MLLP when the clustering option is turned off.

As mentioned above, using the combination of weights and distances in choosing node pairs for matching proved to speed up the algorithm. The rule was tested with several graphs and it performed faster in every case. The difference was found to be from about one second (2-19%) on graphs under 200 nodes to as much as about 110 seconds (about 10%) on graphs with ca. 1400 nodes. The results were especially good with graphs with relatively low density. As no decrease in the layout quality was observed in the images produced by the modified algorithm, it is clear that the modification is useful at least in the implementation using the graph handling functions offered by Cytoscape API.

Table 3: Test results using the original node matching rule and the modified rule.

Graph name	MCL18_m2_178.sif		MCL18_m1_207.sif		AllDetected_642.sif		DE123n1_1406.sif		ptacek.sif	
Number of nodes	178		207		642		1406		1386	
Number of edges	464		6259		5341		19250		4191	
Density	2,61		30,24		8,32		13,69		3.02	
Mean of node degrees	4,88		58,89		15,72		26,58		6.04	
Nodes with degree = 1	62		31		139		107		584	
Nodes with degree <= 5	135		41		380		515		1116	
Nodes with degree <= 10	157		45		466		785		1268	
Nodes with degree <= 50	177		102		555		1167		1359	
Maximum node degree	116		159		100		168		258	
Clustering coefficient	0,23		0,76		0,36		0,33		0.03	
	**Original**	**Modified**	**Original**	**Modified**	**Original**	**Modified**	**Original**	**Modified**	**Original**	**Modified**
Calculation time (sec)	7.55	6.0	5.73	5.64	27.36	24.34	137.797	125.033	1080.801	970.554
Deviation of nodes along x-axis	10,05	9,58	19,66	22,47	40,35	47,46	98,36	94,28	73.97	76.77
Deviation of nodes along y-axis	13,83	11,59	20,7	19,4	45,2	36,79	97,46	88,98	74.6	63.72
Number of edge intersections (sample size)	3322(434)	3076(434)	65999(1000)	65897(1000)	31527(1000)	32978(1000)	24178(1000)	23781(1000)	19069(1000)	18855(1000)
Mean of minimum angles (sample size)	49,29(97)	51,43(97)	1,44(168)	1,65(168)	27,63(415)	25,39(415)	9,71(891)	9,05(891)	23,69(740)	23,14(740)
Edge length standard deviation	21,09	21,11	14,67	16,72	15,1	12,44	14,06	12,57	14.71	14.92

4. Conclusion and future work

In this paper we have described how C. Walshaw's multilevel framework for graph layout can be used within Cytoscape software platform. To enhance our implementation the node matching criterion used by the algorithm was modified to take into account the degrees of the nodes in addition to their weight. We also included an interactive option to enhance the grouping of the nodes using the clustering coefficient values of the graph nodes.

We have tested the implementation of the multilevel framework with several example graphs and compared the results with layouts drawn by several built-in algorithms in Cytoscape. The test results showed, that although being somewhat slower than the other

methods, our implementation was able to generate evenly good quality layouts even for very large graphs. As the tested algorithms use very different methods, it is obvious that the created layouts differ greatly from each other. It is however worth noting that in most cases all the tested algorithms were able to visualize the clusters in the data. As the different layouts methods are easily accessible, it is recommended for a user to study the graph layouts using several different methods. It should also be noted, that certain algorithms may create very low quality layouts. This still promotes the use of several different methods.

An important observation is also that the multilevel framework used in MLLP can be combined with other methods than the force-directed-based layout proposed in the original paper by C. Walshaw. It turns out, that multilevel framework could be used with many different kinds of layout methods to improve or modify the final result. We consider this to be an important aspect for the future development of efficient layout algorithms for Cytoscape.

For the development of open source software products it is vital that there exists a wide scale of open source plug-in-type enhancements. Counting on proprietary libraries and add-ons limits the possibilities of free development of the software. In this way the MLLP also adds value to Cytoscape development although not standing out as the single best layout solution, but to give the users more possibilities and to boost the fitness of Cytoscape for different network types.

References

Brohée , S. & van Helden, J. (2006) Evaluation of clustering algorithms for protein-protein interaction networks. BMC Bioinformatics 2006, 7:488.

Elo, L. L., Järvenpää, H., Matej, O, Lahesmaa, R. & Aittokallio, T. (2007) Systematic construction of gene coexpression networks with applications to human T helper cell differentiation process. Bioinformatics, vol. 23, no.16, 2096-2103.

Fruchterman, T.M.J. and Rheingold, E.M. (1991) Graph drawing by force-directed placement. Software−Practice and Experience, 21, 1129-1164.

Kamada, T. and Kawai, S. (1989) An algorithm for drawing general undirected graphs. Information Processing Letters, 31, 7–15.

Papadimitriou, C. H. and Steiglitz, K. (1982) Combinatorial Optimization: Algorithms and Complexity. Prentice-Hall, Englewood Cliffs, NJ.

Ptacek, J, *et al.* (2005) Global analysis of protein phosphorylation in yeast. Nature 438 (7068): 679-84 .

Purchase, H.C. (2002) Journal of Visual Languages & Computing, Volume 13, Number 5, October 2002 , pp. 501-516 (16)

Shannon, P. *et al.* (2003) Cytoscape: a software environment for integrated models of biomolecular interaction networks. Genome Res., 13, 2498-2504.

Walshaw, C. (2003) A Multilevel Algorithm for Force-Directed Graph-Drawing. Journal of Graph Algorithms and Applications, vol. 7, no. 3, 253 285.

Appendix 1: Test layouts

Figure 1: Graph AllDetected_642 (|V| = 642, |E| = 5341), laid out using (from top to bottom) the MLLP, yFiles Organic layout and Cytoscape Spring Embedded layout.

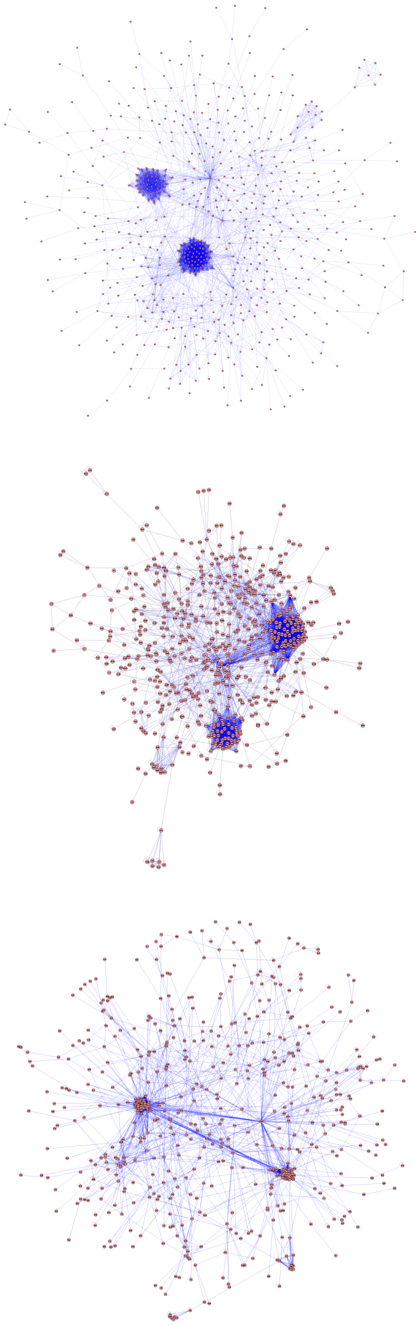

Figure 2: Graph BINF1335_n23_r0.72 (|V| = 884, |E| = 26078), laid out using (from top to bottom) the MLLP, yFiles Organic layout and Cytoscape Spring Embedded layout.

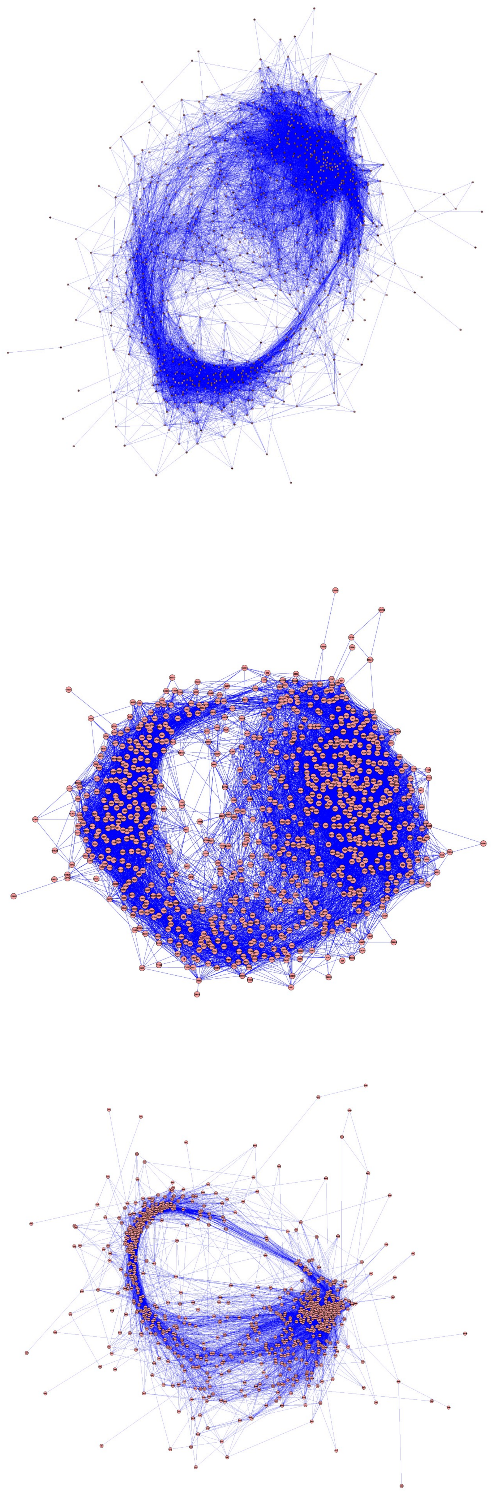

Figure 3: Graph BINF1335_n23_r0.85 (|V| = 757, |E| = 6755), laid out using (from top to bottom) the MLLP, yFiles Organic layout and Cytoscape Spring Embedded layout.

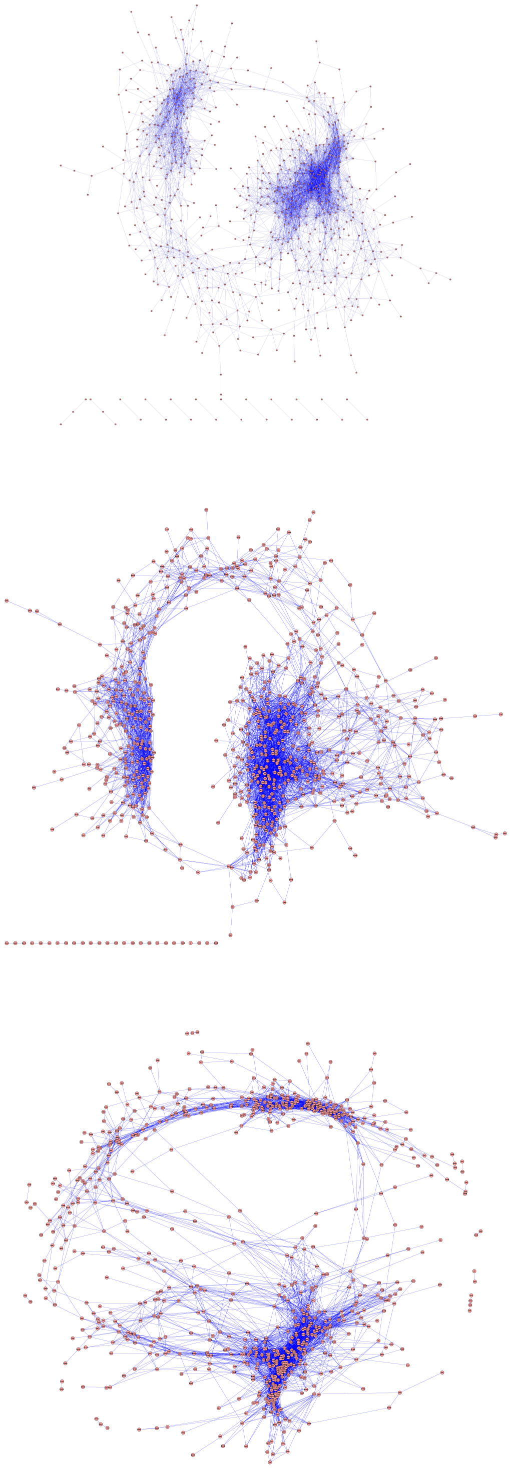

Figure 4: Graph DE123n1_1406 (|V| = 1406, |E| = 19250), laid out using (from top to bottom) the MLLP, yFiles Organic layout and Cytoscape Spring Embedded layout.

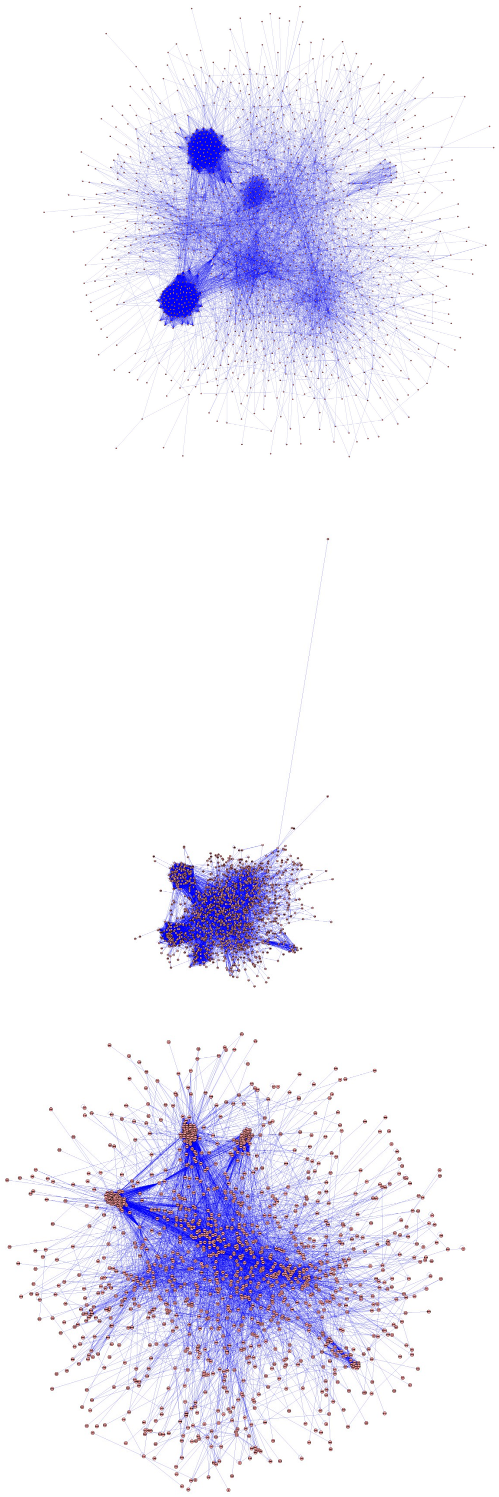

Figure 5: Graph HPRD (|V| = 5699, |E| = 19779), laid out using (from top to bottom) the MLLP, yFiles Organic layout and Cytoscape Spring Embedded layout.

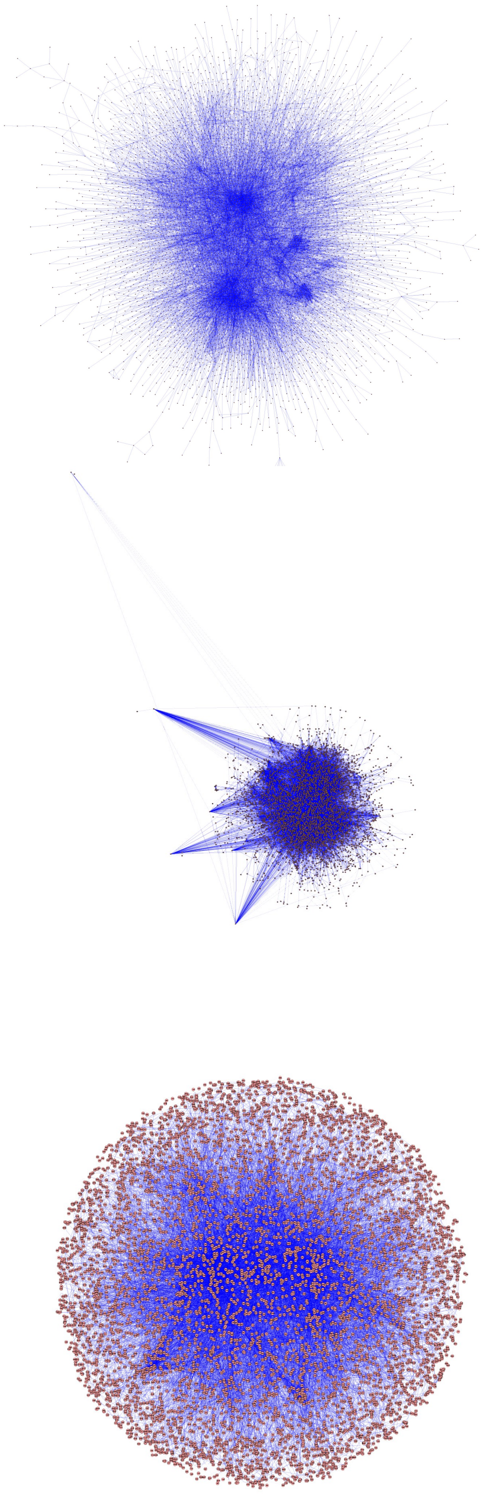

Figure 6: Graph MCL18_m2_178 (|V| = 178, |E| = 464), laid out using (from top to bottom) the MLLP, yFiles Organic layout and Cytoscape Spring Embedded layout.

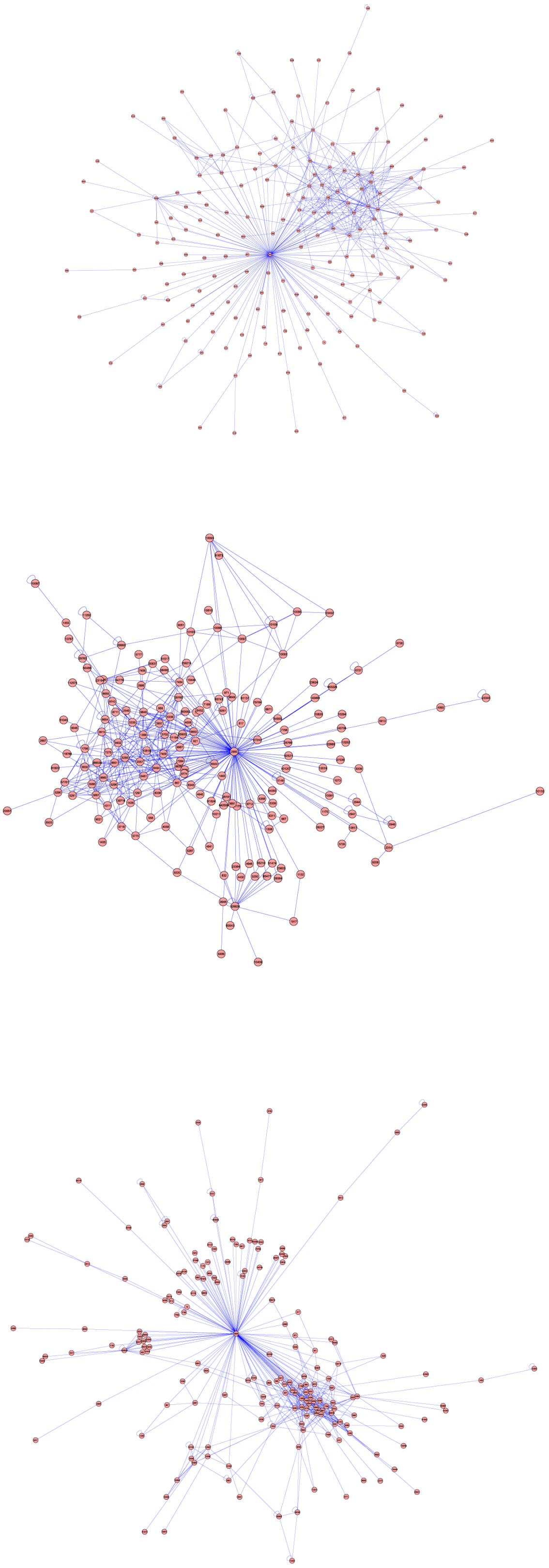

Figure 7: Graph rual (|V| = 2784, |E| = 6438), laid out using (from top to bottom) the MLLP, yFiles Organic layout and Cytoscape Spring Embedded layout.

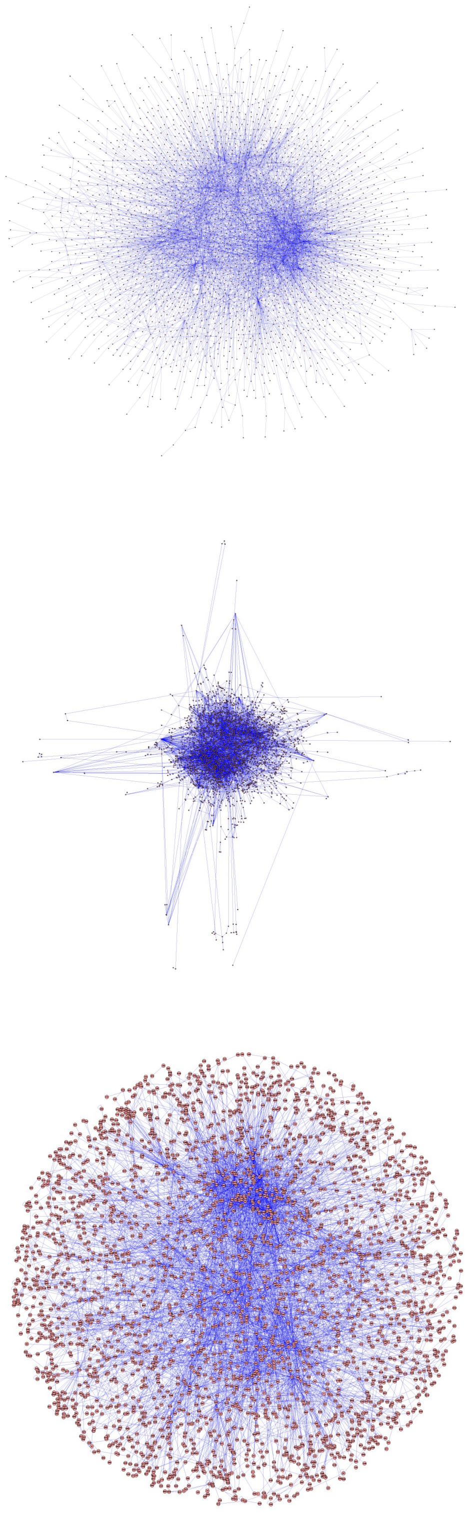

Appendix 2: Test results

**Graph name**	**AllDetected_642.sif**			**BINF1335_n23_r0.72.sif**			**BINF1335_n23_r0.85.sif**			**DE123n1_1406.sif**			**HPRD.sif**			**MCL18_m2_178.sif**			**rual.sif**		
**Layout method**	**MLLP**	**Y-files Organic**	**CS Spring emb.**	**MLLP**	**Y-files Organic**	**CS Spring emb.**	**MLLP**	**Y-files Organic**	**CS Spring emb.**	**MLLP**	**Y-files Organic**	**CS Spring emb.**	**MLLP**	**Y-files Organic**	**CS Spring emb.**	**MLLP**	**Y-files Organic**	**CS Spring emb.**	**MLLP**	**Y-files Organic**	**CS Spring emb.**
**Computation time**	25.11	30.03	17.01	57.39	31	19	21.23	30	20.03	124.25	31	58	1193.01	32	850	6.11	2	2	397.46	30	277
**Deviation of nodes along x-axis**	47,46	46,02	37,92	72,94	56,88	98,6	54,24	64,98	55,49	94,28	106,39	94,06	479,99	796,73	116,44	9,58	12,44	16,38	188,89	461,99	56,54
**Deviation of nodes along y-axis**	36,79	56,71	35	65,84	55,79	112,94	51,6	51,84	49,31	88,98	232,12	92,94	409,44	891,02	124,9	11,59	13,44	12,87	216,18	478,81	59,02
**Number of edge intersections (sample size)**	32978 (1000)	31811 (1000)	29130 (1000)	14752 (1000)	15107 (1000)	16047 (1000)	8222 (1000)	8413 (1000)	8380 (1000)	23781 (1000)	23350 (1000)	25710 (1000)	9191 (1000)	16951 (1000)	17943 (1000)	3076 (434)	2821 (434)	3346 (434)	11494 (1000)	17668 (1000)	14253 (1000)
**Mean of minimum angles (sample size)**	25,39 (415)	29,09 (415)	23,92 (415)	2,15 (834)	3,04 (834)	1,65 (834)	14,33 (601)	13,74 (601)	10,69 (601)	9,05 (891)	14,17 (891)	7,7 (891)	28,71 (793)	30,51 (793)	23,96 (793)	51,43 (97)	51,35 (97)	51,4 (97)	47,36 (838)	47,88 (838)	41,05 (838)
**Edge length standard deviation**	12,44	13,46	12,85	8,89	13,31	6,63	12,69	13,76	7,74	12,57	3,33	13,39	14,37	4,77	15,95	21,11	14,67	20,77	12,23	7,52	15,6

Turku Centre for Computer Science

Joukahaisenkatu 3-5 B, 20520 Turku, Finland | www.tucs.fi

University of Turku
- Department of Information Technology
- Department of Mathematics

Åbo Akademi University
- Department of Information Technologies

Turku School of Economics
- Institute of Information Systems Sciences

ISBN 978-952-12-2022-7
ISSN 1239-1891
