# Supplementary material for: A multilevel layout algorithm for visualizing physical and genetic interaction networks, with emphasis on their modular organization
Source: BioData Min. 2012 Mar 26;5:2. doi: 10.1186/1756-0381-5-2 (PMC3342218; doi:10.1186/1756-0381-5-2)
Supplement: Additional file 2 — User-adjustable settings for the Multilevel Layout plug-in. [file 1756-0381-5-2-S2.PDF]

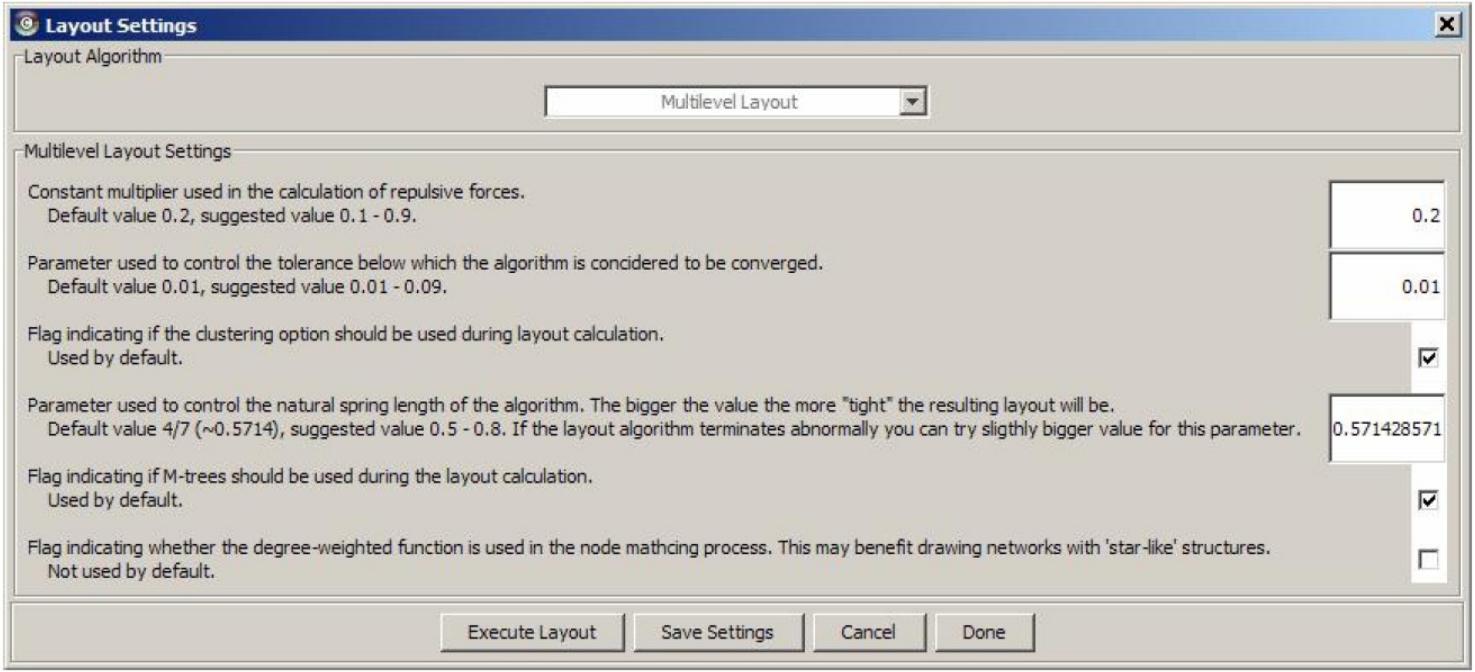

User settings for the Multilevel Layout plug-in, adjustable from the ‘Layout - Settings...” menu of Cytoscape.
