# Supplementary material for: A multilevel layout algorithm for visualizing physical and genetic interaction networks, with emphasis on their modular organization
Source: BioData Min. 2012 Mar 26;5:2. doi: 10.1186/1756-0381-5-2 (PMC3342218; doi:10.1186/1756-0381-5-2)
Supplement: Additional file 3 — Comparison of the layout algorithms using semantic similarity. [file 1756-0381-5-2-S3.PDF]

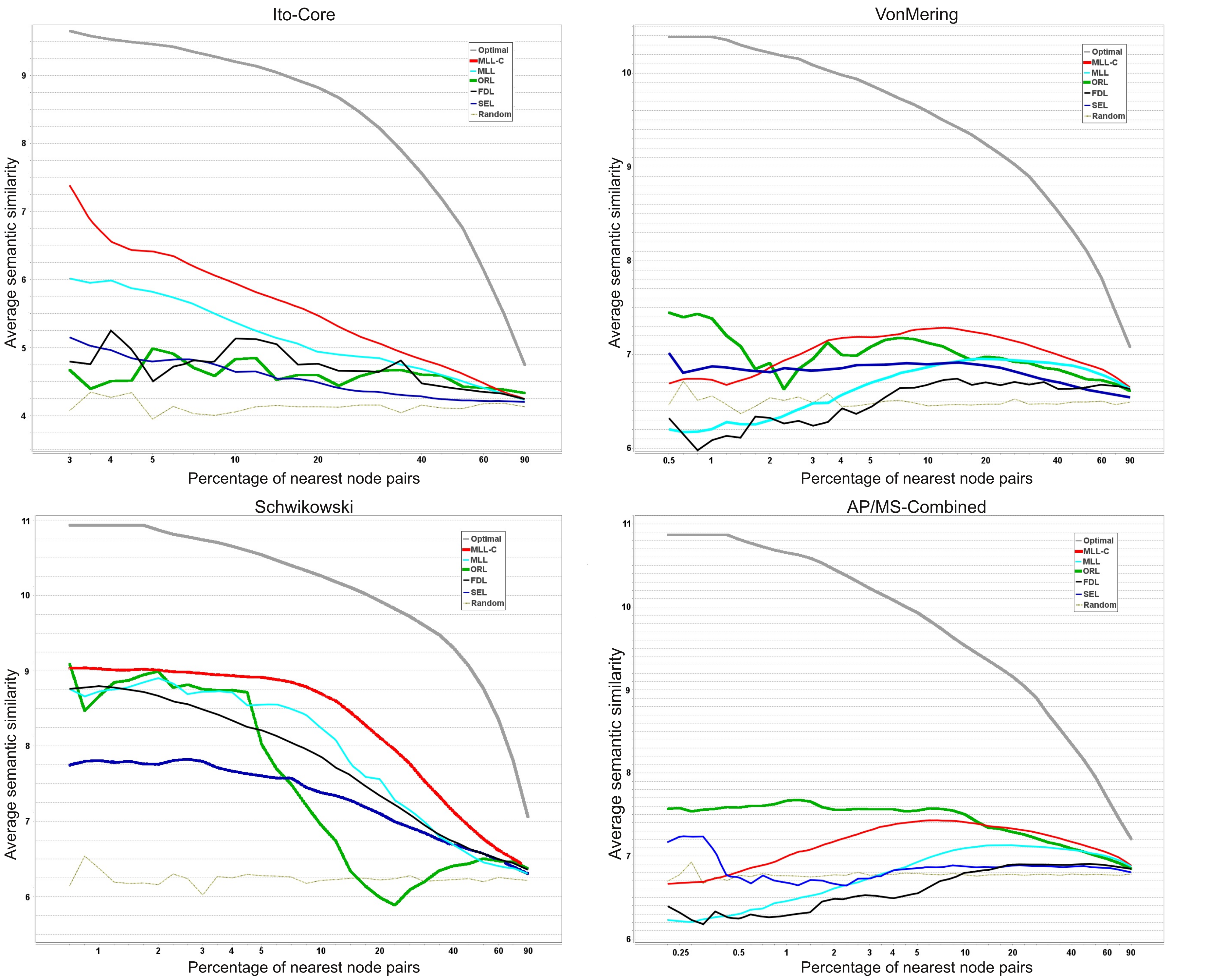

Comparison of the layout algorithms using semantic similarity in the test networks: Ito-Core, VonMering, Schwikowski, and AP/MS-Combined. The x-axis (logarithmic scale) gives the percentage of the nearest node pairs (the connected pairs having the smallest Euclidean distance in the particular layout), and the y-axis shows the average semantic similarity among these pairs (agreement in the biological process terms annotated with those nodes in GO).

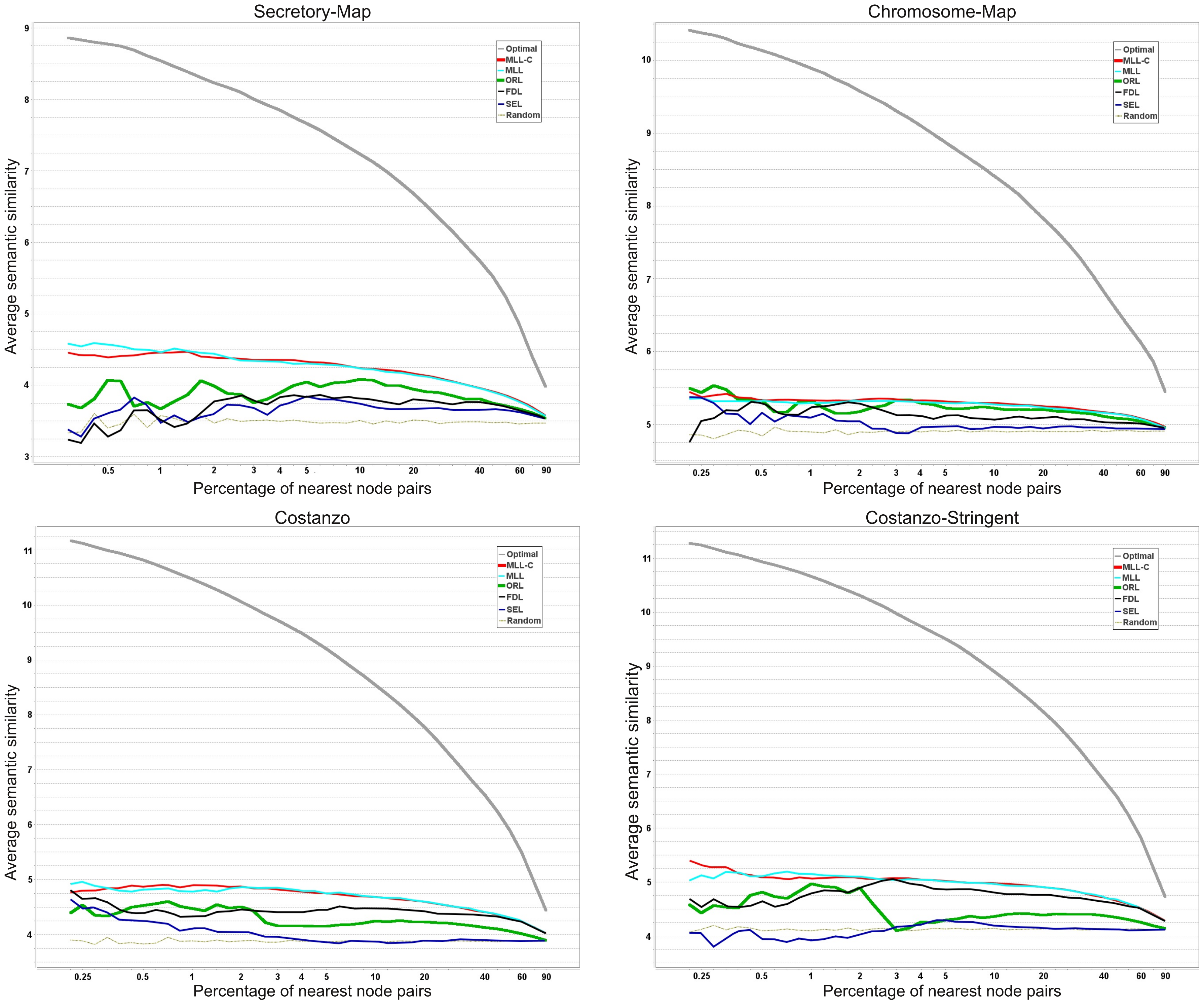

Comparison of the layout algorithms using semantic similarity in the test networks: Secretory-Map, Chromosome-Map, Costanzo, and Costanzo-Stringent. The x-axis (logarithmic scale) gives the percentage of the nearest node pairs (the connected pairs having the smallest Euclidean distance in the particular layout), and the y-axis shows the average semantic similarity among these pairs (agreement in the biological process terms annotated with those nodes in GO).

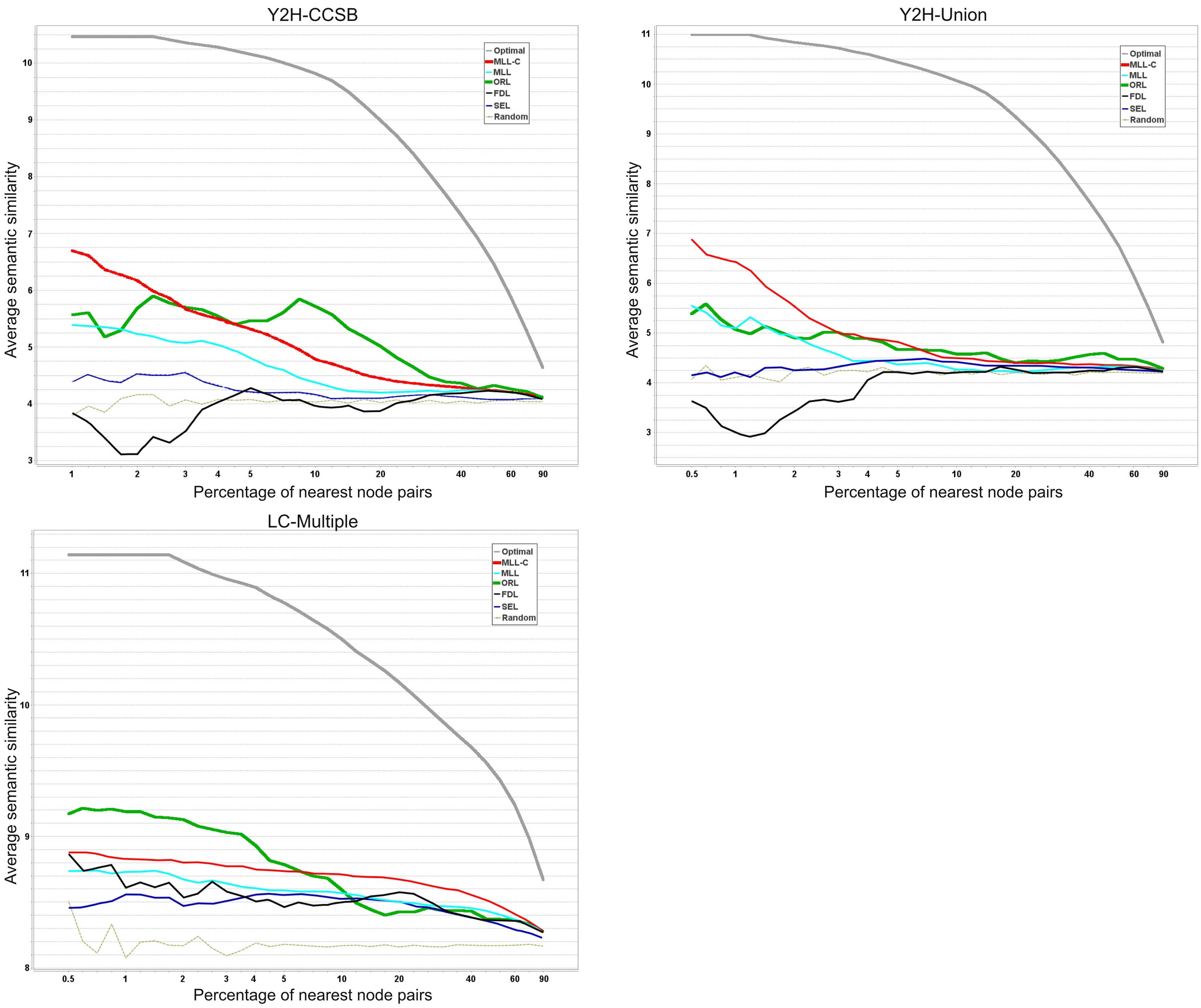

Comparison of the layout algorithms using semantic similarity in the test networks: Y2H-CCSB, Y2H-Union, and LC-Multiple. The x-axis (logarithmic scale) gives the percentage of the nearest node pairs (the connected pairs having the smallest Euclidean distance in the particular layout), and the y-axis shows the average semantic similarity among these pairs (agreement in the biological process terms annotated with those nodes in GO).
