# Supplementary material for: A multilevel layout algorithm for visualizing physical and genetic interaction networks, with emphasis on their modular organization
Source: BioData Min. 2012 Mar 26;5:2. doi: 10.1186/1756-0381-5-2 (PMC3342218; doi:10.1186/1756-0381-5-2)
Supplement: Additional file 4 — Running times and semantic similarities for the test networks. [file 1756-0381-5-2-S4.PDF]

Test networks			Network parameters						Running Time (s)									
**Network**	**Type**	**Screen**	**Nodes**	**Edges**	**Density**	**MND**	**MND_SD**	**MCC**	**MLL**	**SEM**	**MLL-C**	**SEM**	**FDL**	**SEM**	**SEL**	**SEM**	**ORL**	**SEM**
Ito-Core	PI	PPI	426	568	0.006	2.667	3.919	0.093	3.100	0.043	3.600	0.044	0.600	0.008	3.700	0.042	1.100	0.000
VonMering	PI	PPI	573	2097	0.013	7.319	9.017	0.450	3.000	0.054	3.400	0.028	1.000	0.010	90.900	1.772	6.300	0.000
Schwikowski	PI	PPI	1297	1862	0.002	2.871	3.109	0.125	12.900	0.069	13.800	0.171	3.300	0.009	32.200	0.254	30.600	0.000
Y2H-CCSB	PI	PPI	964	1598	0.003	3.315	5.456	0.095	12.200	0.098	13.400	0.089	2.100	0.010	37.900	0.284	21.300	0.000
Y2H-Union	PI	PPI	1647	2682	0.002	3.257	5.334	0.086	30.500	0.262	30.700	0.230	5.100	0.035	101.500	0.339	28.500	0.000
AP/MS-Combined	PI	CCA	1004	8319	0.017	16.571	18.627	0.648	6.660	0.072	8.200	0.063	2.400	0.015	N/A	N/A	30.800	0.000
LC-Multiple	MT	LCI	1213	2621	0.004	4.322	4.533	0.337	9.000	0.104	10.000	0.086	3.000	0.017	77.800	1.512	30.900	0.000
Secretory-Map	GI	E-MAP	409	4175	0.050	20.416	23.824	0.251	4.200	0.062	5.000	0.051	0.700	0.009	775.000	0.000	3.600	0.000
Chromosome-Map	GI	E-MAP	735	17185	0.064	46.762	43.609	0.233	17.300	0.142	22.300	0.154	1.900	0.114	N/A	N/A	26.300	0.000
Costanzo	GI	SGA	4319	74984	0.007	29.956	41.858	0.062	326.500	2.912	388.400	10.773	29.900	2.417	N/A	N/A	31.400	0.000
Costanzo-Stringent	GI	SGA	3811	35924	0.004	16.067	22.918	0.046	207.900	1.320	231.300	3.725	21.400	0.462	N/A	N/A	29.900	0.000

Test networks			Network parameters						Semantic similarity score									
**Network**	**Type**	**Screen**	**Nodes**	**Edges**	**Density**	**MND**	**MND_SD**	**MCC**	**MLL**	**SEM**	**MLL-C**	**SEM**	**FDL**	**SEM**	**SEL**	**SEM**	**ORL**	**SEM**
Ito-Core	PI	PPI	426	568	0.006	2.667	3.919	0.093	0.237	0.011	0.337	0.009	0.137	0.000	0.098	0.014	0.110	0.000
VonMering	PI	PPI	573	2097	0.013	7.319	9.017	0.450	0.050	0.006	0.175	0.006	-0.004	0.000	0.108	0.023	0.169	0.000
Schwikowski	PI	PPI	1297	1862	0.002	2.871	3.109	0.125	0.406	0.005	0.536	0.001	0.442	0.000	0.280	0.017	0.326	0.000
Y2H-CCSB	PI	PPI	964	1598	0.003	3.315	5.456	0.095	0.008	0.008	0.204	0.006	-0.026	0.000	0.040	0.013	0.221	0.000
Y2H-Union	PI	PPI	1647	2682	0.002	3.257	5.334	0.086	0.066	0.007	0.144	0.007	-0.052	0.000	0.023	0.015	0.103	0.010
AP/MS-Combined	PI	CCA	1004	8319	0.017	16.571	18.627	0.648	-0.004	0.003	0.121	0.004	-0.062	0.000	N/A	N/A	0.220	0.001
LC-Multiple	MT	LCI	1213	2621	0.004	4.322	4.533	0.337	0.174	0.005	0.228	0.004	0.156	0.000	0.131	0.013	0.252	0.001
Secretory-Map	GI	E-MAP	409	4175	0.050	20.416	23.824	0.251	0.199	0.006	0.195	0.007	0.050	0.000	0.043	0.000	0.100	0.000
Chromosome-Map	GI	E-MAP	735	17185	0.064	46.762	43.609	0.233	0.095	0.004	0.101	0.004	0.059	0.000	N/A	N/A	0.087	0.000
Costanzo	GI	SGA	4319	74984	0.007	29.956	41.858	0.062	0.159	0.004	0.160	0.010	0.106	0.000	N/A	N/A	0.080	0.000
Costanzo-Stringent	GI	SGA	3811	35924	0.004	16.067	22.918	0.046	0.165	0.008	0.166	0.005	0.120	0.000	N/A	N/A	0.070	0.000

Running times (upper table) and semantic similarity scores (lower table) for the test networks generated with the layout algorithms. N/A, running time was longer than one hour. SEM, standard error of the mean over the replicate layout runs.Highlighted is the best algorithm for each test network in terms of the computation time or semantic similarity. Network types: PI, physical interactions; GI, genetic interactions; MT, mixed type. Screening methods: PPI, protein-protein interaction; CCA, protein co-complex association; LCI, literate-curated interactions; E-MAP, epistatic miniarray profiling; SGA, synthetic genetic array mapping. Topological network parameters: MND, mean node degree; MND_SD,standard deviation of MND; MCC, mean clustering coefficient of the network. Costanzo-Stringent was extracted using the interaction score cut-offs $\varepsilon<-0.17$ or $\varepsilon>0.21$.
