# Supplementary material for: A multilevel layout algorithm for visualizing physical and genetic interaction networks, with emphasis on their modular organization
Source: BioData Min. 2012 Mar 26;5:2. doi: 10.1186/1756-0381-5-2 (PMC3342218; doi:10.1186/1756-0381-5-2)
Supplement: Additional file 5 — Multiple runs of the MLL-C algorithm in the Ito-Core network. [file 1756-0381-5-2-S5.PDF]

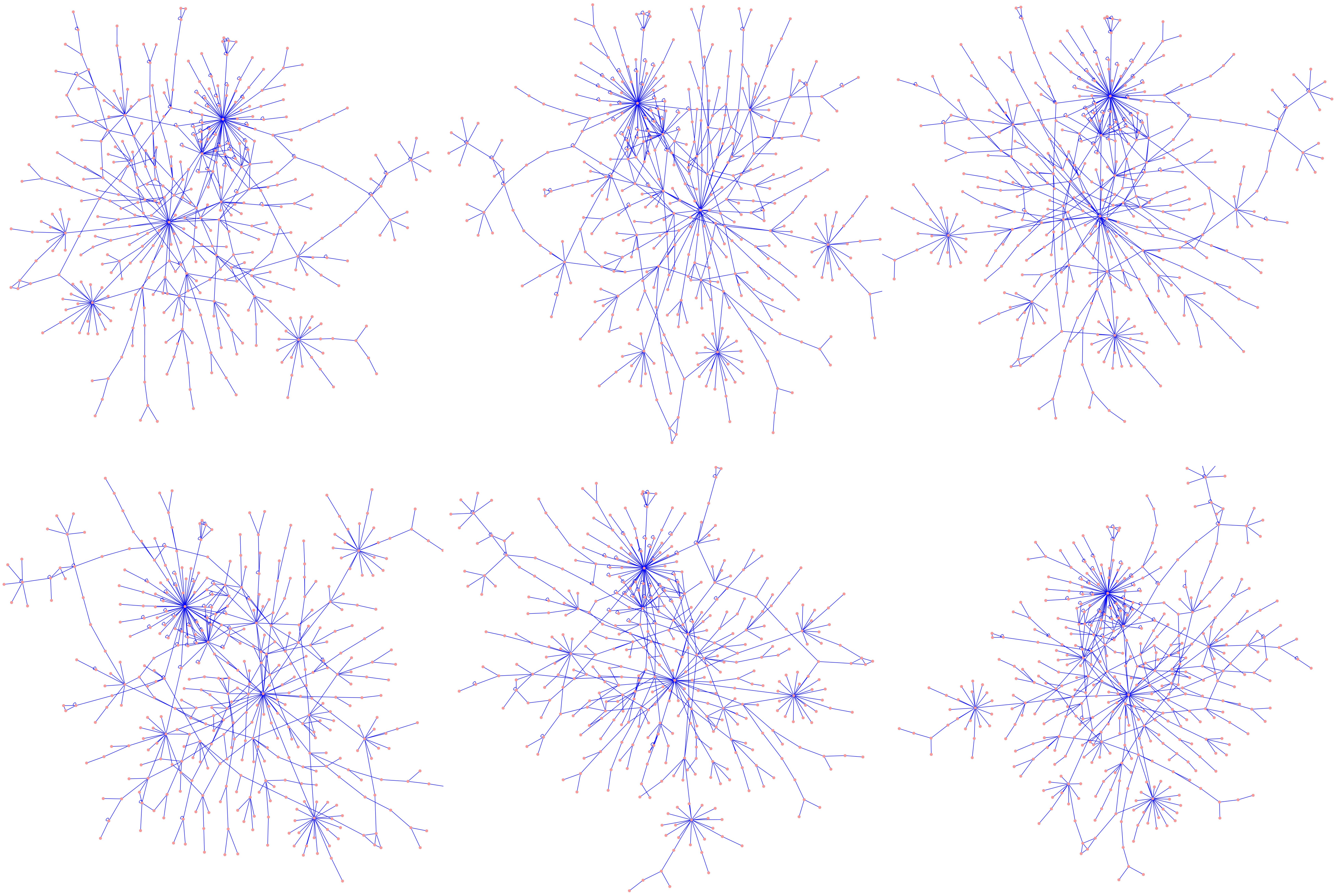

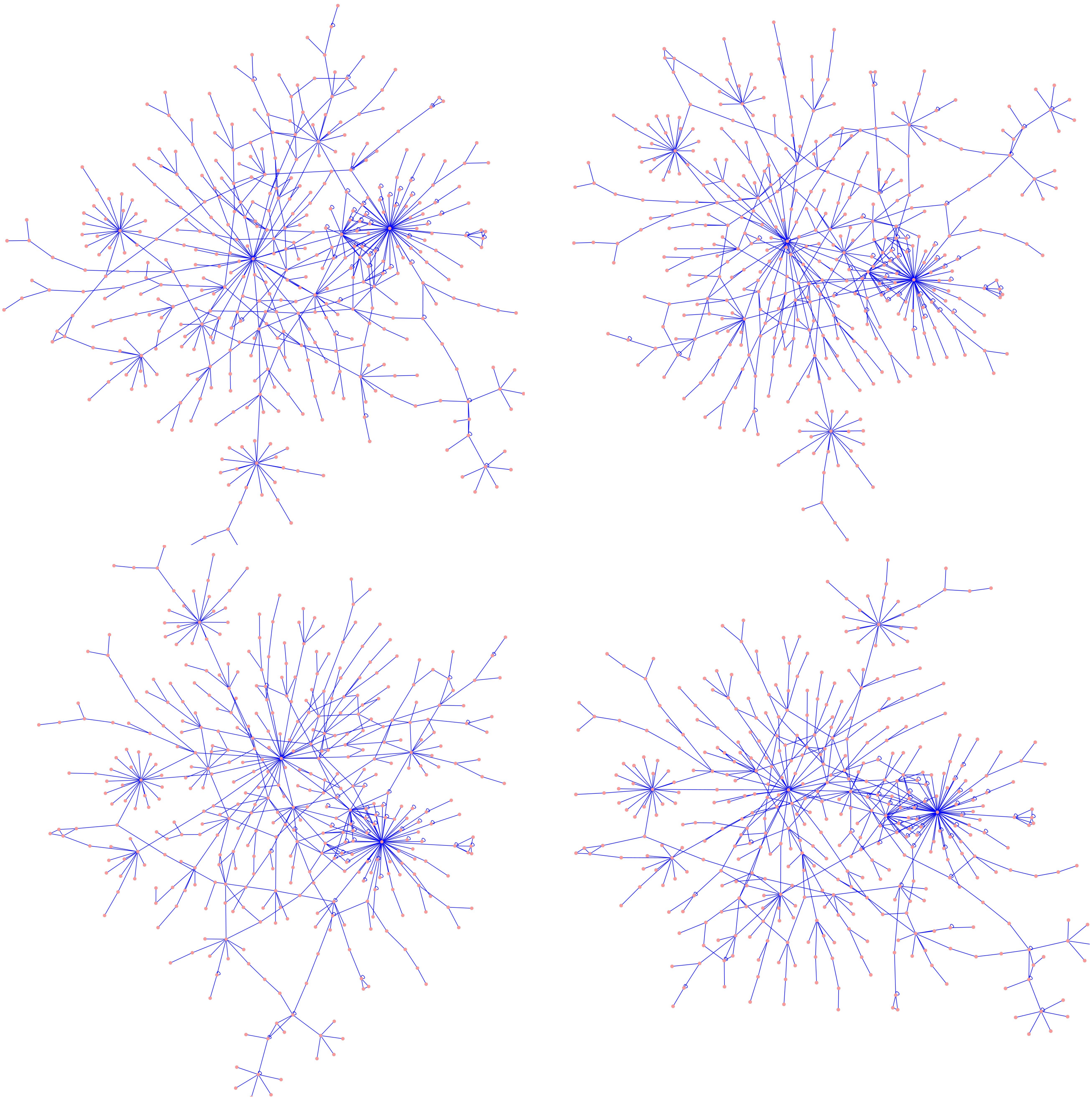

Visualization of ten different initializations of the MLL-C algorithm in the Ito-Core network, demonstrating how the initial layout affects the final layout solution. Ito-core network was used as an example since it has visually distinguishable features, such as network clusters, which allow comparison of the different runs. The network also resulted in a relatively large variation in the semantic similarity over the multiple initializations (Additional File 3).
