# Supplementary material for: A multilevel layout algorithm for visualizing physical and genetic interaction networks, with emphasis on their modular organization
Source: BioData Min. 2012 Mar 26;5:2. doi: 10.1186/1756-0381-5-2 (PMC3342218; doi:10.1186/1756-0381-5-2)
Supplement: Additional file 6 — Correlation coefficients for running times and semantic scores. [file 1756-0381-5-2-S6.PDF]

	Network parameters						Running time					Semantic score					
	Nodes	Edges	Density	MND	MND SD	AVG CC	MLL	MLL-C	FDL	SEL	ORL	MLL	MLL-C	FDL	SEL	ORL	
**Nodes**		0.886	-0.363	0.233	0.436	-0.451	0.960	0.952	0.982	-0.423	0.566	0.082	-0.170	0.130	0.179	-0.351	**Network parameters**
**Edges**	0.886		-0.043	0.550	0.731	-0.327	0.968	0.975	0.949	0.837	0.393	0.017	-0.294	0.041	-0.272	-0.485	
**Density**	-0.363	-0.043		0.772	0.634	0.231	-0.226	-0.214	-0.277	0.974	-0.254	-0.112	-0.371	-0.199	-0.317	-0.370	
**MND**	0.233	0.550	0.772		0.966	0.053	0.365	0.377	0.320	0.980	0.181	-0.161	-0.515	-0.153	-0.312	-0.528	
**MND SD**	0.436	0.731	0.634	0.966		-0.035	0.575	0.586	0.530	0.978	0.211	-0.150	-0.533	-0.159	-0.396	-0.601	
**AVG CC**	-0.451	-0.327	0.231	0.053	-0.035		-0.441	-0.432	-0.438	0.206	-0.049	-0.424	-0.288	-0.349	0.068	0.336	
**MLL**	0.960	0.968	-0.226	0.365	0.575	-0.441		0.999	0.995	-0.237	0.394	0.072	-0.216	0.084	-0.187	-0.469	**Running time**
**MLL-C**	0.952	0.975	-0.214	0.377	0.586	-0.432	0.999		0.992	-0.237	0.389	0.070	-0.217	0.084	-0.180	-0.465	
**FDL**	0.982	0.949	-0.277	0.320	0.530	-0.438	0.995	0.992		-0.350	0.452	0.080	-0.200	0.103	0.095	-0.430	
**SEL**	-0.423	0.837	0.974	0.980	0.978	0.206	-0.237	-0.237	-0.350		-0.414	0.051	-0.290	-0.192	-0.341	-0.438	
**ORL**	0.566	0.393	-0.254	0.181	0.211	-0.049	0.394	0.389	0.452	-0.414		-0.042	-0.091	0.171	0.346	0.251	
**MLL**	0.082	0.017	-0.112	-0.161	-0.150	-0.424	0.072	0.070	0.080	0.051	-0.042		0.844	0.939	0.801	0.248	**Semantic score**
**MLL-C**	-0.170	-0.294	-0.371	-0.515	-0.533	-0.288	-0.216	-0.217	-0.200	-0.290	-0.091	0.844		0.866	0.890	0.608	
**FDL**	0.130	0.041	-0.199	-0.153	-0.159	-0.349	0.084	0.084	0.103	-0.192	0.171	0.939	0.866		0.943	0.447	
**SEL**	0.179	-0.272	-0.317	-0.312	-0.396	0.068	-0.187	-0.180	0.095	-0.341	0.346	0.801	0.890	0.943		0.799	
**ORL**	-0.351	-0.485	-0.370	-0.528	-0.601	0.336	-0.469	-0.465	-0.430	-0.438	0.251	0.248	0.608	0.447	0.799		

	Nodes	Edges	Density	MND	MND_SD	AVG CC	MLL	MLL-C	FDL	SEL	ORL	MLL	MLL-C	FDL	SEL	ORL	
**Nodes**		2.776E-04	0.272	0.490	0.180	0.164	2.808E-06	6.273E-06	8.248E-08	0.195	0.070	0.811	0.617	0.703	0.597	0.290	**Network parameters**
**Edges**	0.000		0.901	0.080	0.011	0.326	1.025E-06	3.730E-07	8.336E-06	1.318E-03	0.231	0.960	0.379	0.906	0.419	0.130	
**Density**	0.272	0.901		0.005	0.036	0.494	0.505	0.527	0.410	4.392E-07	0.451	0.742	0.261	0.558	0.342	0.263	
**MND**	0.490	0.080	0.005		1.393E-06	0.876	0.270	0.253	0.338	1.391E-07	0.595	0.637	0.105	0.654	0.350	0.095	
**MND SD**	0.180	0.011	0.036	0.000		0.918	0.064	0.058	0.093	1.955E-07	0.534	0.659	0.091	0.640	0.228	0.050	
**AVG CC**	0.164	0.326	0.494	0.876	0.918		0.175	0.185	0.177	0.543	0.887	0.193	0.390	0.293	0.843	0.312	
**MLL**	0.000	0.000	0.505	0.270	0.064	0.175		2.638E-14	1.681E-10	0.483	0.231	0.834	0.523	0.805	0.582	0.146	**Running time**
**MLL-C**	0.000	0.000	0.527	0.253	0.058	0.185	0.000		1.717E-09	0.482	0.238	0.837	0.522	0.807	0.597	0.150	
**FDL**	0.000	0.000	0.410	0.338	0.093	0.177	0.000	0.000		0.291	0.162	0.816	0.555	0.763	0.781	0.187	
**SEL**	0.195	0.001	0.000	0.000	0.000	0.543	0.483	0.482	0.291		0.205	0.882	0.386	0.572	0.305	0.178	
**ORL**	0.070	0.231	0.451	0.595	0.534	0.887	0.231	0.238	0.162	0.205		0.903	0.789	0.616	0.297	0.457	
**MLL**	0.811	0.960	0.742	0.637	0.659	0.193	0.834	0.837	0.816	0.882	0.903		1.084E-03	1.800E-05	3.046E-03	0.461	**Semantic score**
**MLL-C**	0.617	0.379	0.261	0.105	0.091	0.390	0.523	0.522	0.555	0.386	0.789	0.001		5.603E-04	2.391E-04	0.047	
**FDL**	0.703	0.906	0.558	0.654	0.640	0.293	0.805	0.807	0.763	0.572	0.616	0.000	0.001		1.354E-05	0.168	
**SEL**	0.597	0.419	0.342	0.350	0.228	0.843	0.582	0.597	0.781	0.305	0.297	0.003	0.000	0.000		0.003	
**ORL**	0.290	0.130	0.263	0.095	0.050	0.312	0.146	0.150	0.187	0.178	0.457	0.461	0.047	0.168	0.003		

Pearson correlation coefficients (upper table), and their statistical significance (lower table, from *t*-distribution), among the network parameters, running times and semantic scores. Highlighted are significant correlations ($p<0.05$)
