# Supplementary material for: A multilevel layout algorithm for visualizing physical and genetic interaction networks, with emphasis on their modular organization
Source: BioData Min. 2012 Mar 26;5:2. doi: 10.1186/1756-0381-5-2 (PMC3342218; doi:10.1186/1756-0381-5-2)
Supplement: Additional file 7 — Relative speed-up of MLL-C provided by the M-tree architecture. [file 1756-0381-5-2-S7.PDF]

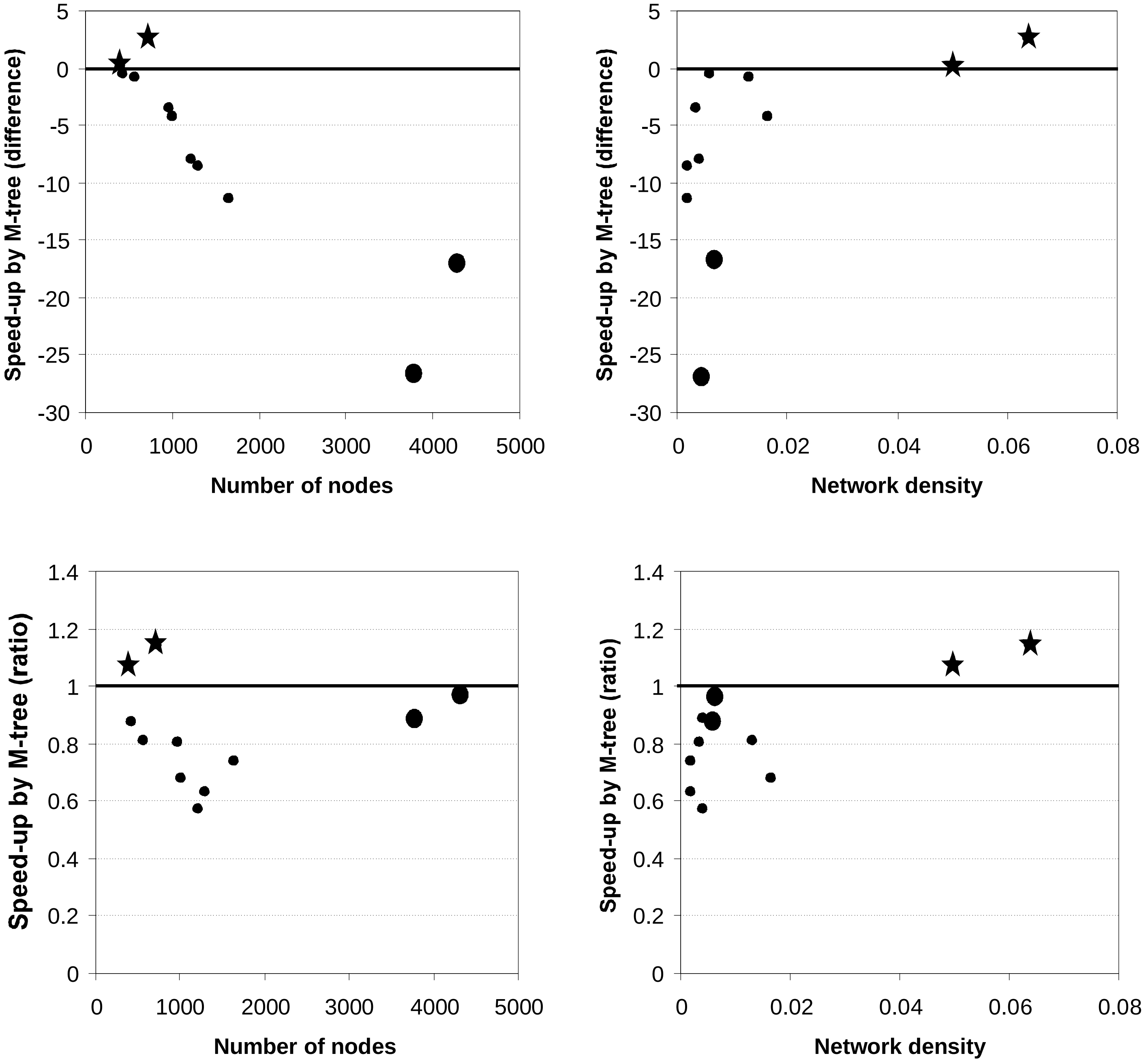

The relative speed-up of the MLL-C algorithm provided by M-tree architecture, shown both in the absolute running time difference (upper graphs), as well as in the proportion of the running times with and without the M-tree architecture (lower graphs). The M-tree provided largest benefits in larger network (left), which have only a moderately dense connection structure (right). The stars indicate the two densest networks (Secretory and Chromosome E-MAP genetic interaction networks), in which the M-tree is in fact ineffective. The larger points indicate the two largest networks (Costanzo and Costanzo-Stringent SGA genetic interaction networks), in which the M-trees decrease the absolute running times but not the proportional.
