# Supplementary material for: A multilevel layout algorithm for visualizing physical and genetic interaction networks, with emphasis on their modular organization
Source: BioData Min. 2012 Mar 26;5:2. doi: 10.1186/1756-0381-5-2 (PMC3342218; doi:10.1186/1756-0381-5-2)
Supplement: Additional file 8 — Layout solutions when applied to the human HPRD network. [file 1756-0381-5-2-S8.PDF]

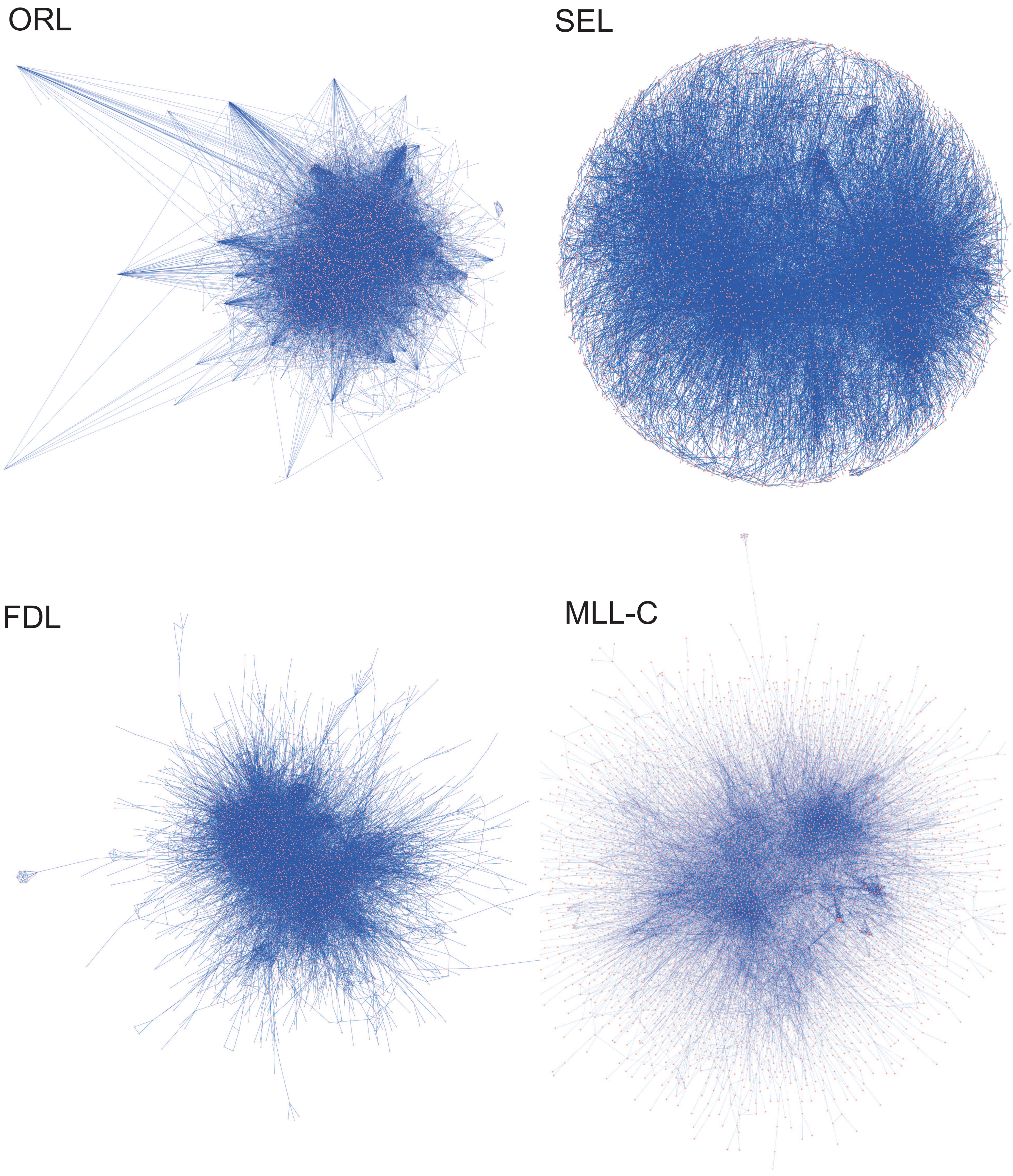

Layout solutions using the yFiles Organic layout (ORL), Cytoscape's Spring-embedded layout (SEL) and Force-directed layout (FDL), or Multilevel layout with the clustering option (MLL-C), when applied to the Human Protein Reference Database (HPRD) network in Cytoscape, which consists of 5699 protein nodes and 19779 literature-curated protein-protein interactions. The node sizes and edge widths were standardized in Cytoscape to make the layout displays comparable in accuracy (the same zooming resolution was used in the export). The running times of the layout algorithms were as follows: YOL 1 minute, SEL 20 minutes, FDL 2 minutes, and MLL-C 8 minutes on a desktop Linux machine and Cytoscape version 2.8.0.
