# Supplementary material for: A multilevel layout algorithm for visualizing physical and genetic interaction networks, with emphasis on their modular organization
Source: BioData Min. 2012 Mar 26;5:2. doi: 10.1186/1756-0381-5-2 (PMC3342218; doi:10.1186/1756-0381-5-2)
Supplement: Additional file 9 — The top-scoring clusters found in the Schwikowski network. [file 1756-0381-5-2-S9.PDF]

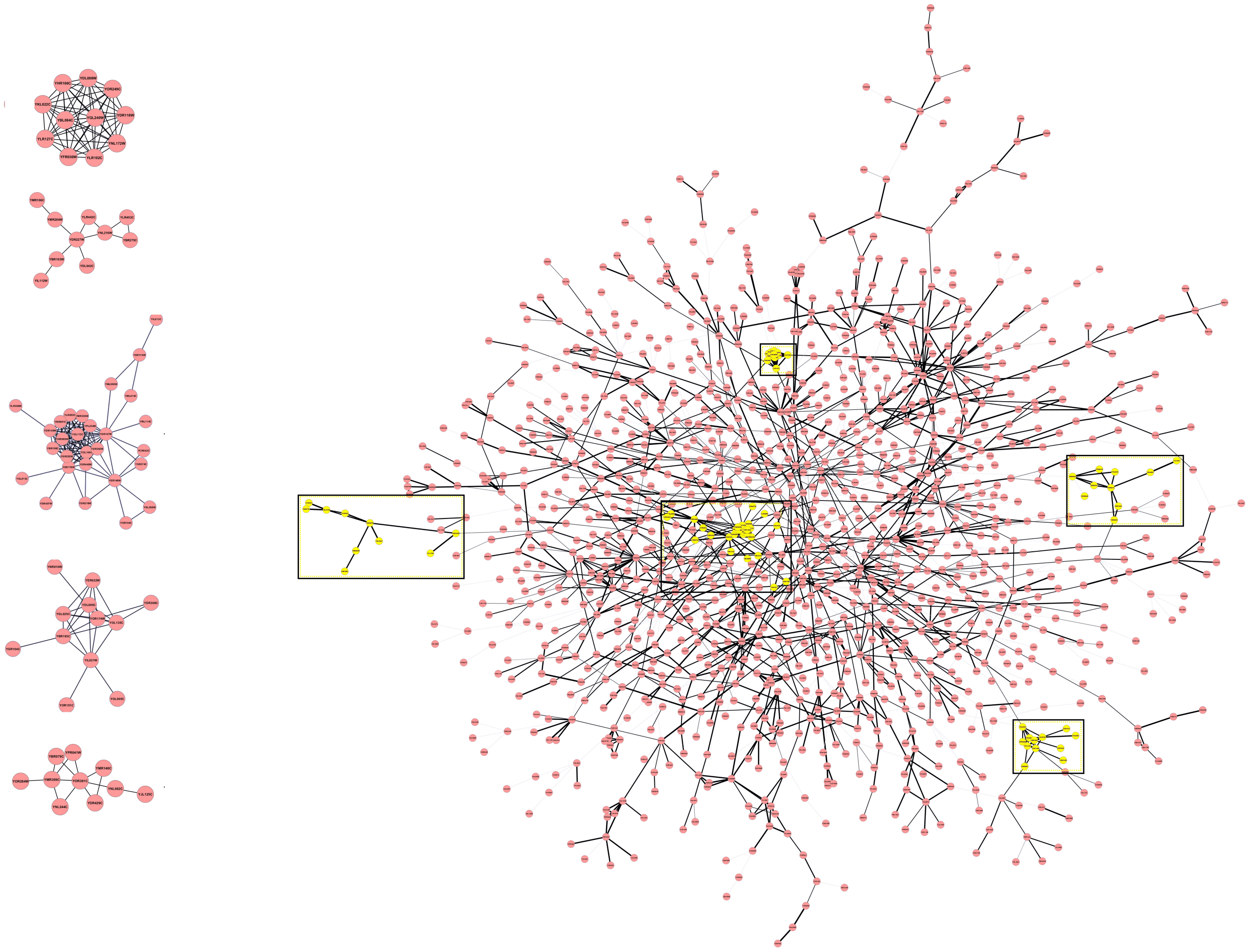

The top-scoring clusters (individual layouts on the left) found in the Schwikowski network (the boxes inside the overall layout on the right) using the MLL-C and ClusterViz plug-ins (http://code.google.com/p/clusterviz-cytoscape/). The edges were weighted and shaded according to their GO biological process semantic similarity. The network clusters were subsequently searched for enrichment of known protein complexes in yeast using the curated information provided by EMBL (http://yeast-complexes.russelllab.org). For instance, all of the nodes of the top-scoring cluster belong to the Anaphase-promoting complex (APC or cyclosome), which is an essential ubiquitin protein ligase that regulates mitotic progression and exit by controlling the stability of cell cycle regulatory proteins, such as securin and the mitotic cyclins.
